# *Lactiplantibacillus plantarum* D25: A Promising Probiotic with Multi-Target Antibacterial Efficacy, Unique Metabolic Traits, Dual Thermal Sensitivity, and Biopreservative Potential for Refrigerated Shrimp

**DOI:** 10.3390/foods15213951

**Published:** 2026-11-03

**Authors:** Tong Yang, Yongxin Yu, Yongjie Wang, Lu Xie, Bailin Li, Lanming Chen

**Affiliations:** 1 College of Food Science and Technology, Shanghai Ocean University, Shanghai 201306, China; 2 Shanghai–MOST Key Laboratory of Health and Disease Genomics, Shanghai Institute for Biomedical and Pharmaceutical Technologies, Shanghai 200237, China

**Keywords:** *Lactiplantibacillus plantarum* D25, antibacterial activity, biopreservation, probiotic properties, biosafety, shrimp preservation

## Abstract

The improper use of chemical preservatives poses significant food safety risks, highlighting the urgent need for safer and more sustainable preservation methods. In this study, we systematically evaluated antibacterial efficacy, probiotic properties, and biosafety of a novel *Lactiplantibacillus plantarum* D25 strain (3.29 Mb) through comprehensive phenotypic and genotypic analyses. The cell-free supernatant (CFS) of *L. plantarum* D25 (CFS-*Lp*D25) demonstrated broad-spectrum antimicrobial activity, with particularly strong inhibition on *Vibrio cholerae,* yielding an inhibition zone diameter of 20.96 mm. The potent antibacterial effects were primarily attributed to the low pH (3.83) and high levels of organic acids such as lactic acid (12.49 mg/mL). Application of 10% CFS-*Lp*D25 significantly prolonged the refrigerated shelf life of Pacific white shrimp (*Litopenaeus vannamei*) meat, surpassing the effectiveness of the chemical preservative potassium sorbate. This treatment also effectively mitigated sensory deterioration, stabilized pH, and reduced lipid oxidation and protein degradation. *L. plantarum* D25 exhibited tolerance to gastrointestinal and food processing stresses, thriving at pH 3.5, under osmotic pressure (≤4% NaCl), and in bile salts (<20 mM). Its dual thermal sensitivity (50–55 °C) enhanced its suitability for industrial applications, with unique metabolic traits. Biosafety assessments confirmed the absence of hemolytic and gelatinase activities. Overall, these findings establish *L. plantarum* D25 as a potential probiotic candidate and natural biopreservative in functional foods with antibacterial activity, supporting the reduction in chemical preservatives in the food industry.

## 1. Introduction

The *Lactiplantibacillus* spp. including *Lactiplantibacillus plantarum* are commonly found in fermented dairy, meat, and vegetable products, as well as in the gastrointestinal tract of humans and animals [1,2]. Owing to their favorable probiotic properties, *Lactiplantibacillus* spp. are widely used in the food industry (e.g., yogurt, and kimchi), animal feed production, and health care sectors [3,4]. Numerous studies have indicated that *Lactiplantibacillus* spp. could enhance flavor and nutrition, extending food shelf life; improve animal digestion, absorption and immunity; and regulate intestinal health, alleviating inflammatory bowel disease, and ameliorating metabolic syndrome [5,6,7].

The first complete genome of *L. plantarum* WCFS1 (3.31 Mb, GenBank: GCA_000203855.3) was sequenced in 2003 [8]. With advancements in sequencing technologies, 1671 complete *L. plantarum* genomes (not including incomplete or low-quality genomes) are available in the GenBank database so far, ranging from 2.5 to 3.8 Mb, with an average G+C content of approximately 45%. This wealth of genomic data has enabled the identification of many probiotic-associated genes in *L. plantarum* strains. For example, Arjun et al. [9] isolated the *L. plantarum* ILSF15 strain from gut microbiota of tribal populations in Odisha, India, in 2019. Genomic analysis revealed that this strain (3.30 Mb) carries genes involved in the biosynthesis of various bioactive compounds, such as plantaricin E/F, vitamin K2, and vitamin B6 [9]. Gizachew & Engidawork reported that *L. plantarum* 54A (3.29 Mb), 54B (3.39 Mb), and 54C (3.37 Mb) strains, isolated from Ethiopian traditional fermented cottage cheese, possess genes encoding key carbohydrate-related biosynthetic enzymes, gene clusters for lactone autoinducers, terpenoids, and type III polyketide synthases, as well as complete gene sets required for glycopeptide and riboflavin biosynthesis [10].

In addition to probiotic-associated genes, *L. plantarum* genomes may contain potential risk factors, such as virulence determinants and antibiotic resistance (AR) genes (ARGs). For instance, many *L. plantarum* strains have demonstrated resistance to aminoglycoside antibiotics, including gentamicin (GEN) and kanamycin (KAN) [11,12,13]. Recently, Keter et al. [14] isolated *Lactobacillus* strains (*n* = 53) from various food samples in Egypt in 2020 and assessed their virulence factors and AR profiles. They found that *Lactobacillus brevis* MIM04 showed resistance to vancomycin (VAN), ampicillin (AMP), cefotaxime (CTX), oxacillin (OXA), and GEN [14]. Pswarayi et al. analyzed *Lactobacillus* strains (*n* = 54) from a Zimbabwean fermented cereal beverage and found that some strains acquired multidrug resistance (MDR)-associated genes through horizontal gene transfer (HGT) [15]. *L. plantarum* strains show significant variation in AR profiles and may transfer ARGs within the gut if they possess mobile genetic elements (MGEs) [16,17], posing health risks to hosts.

Given the broad commercial potential and possible safety risks of *Lactiplantibacillus*, selecting effective biosafety assessment methods is essential for its application in promoting gut health and its exploration as probiotic or novel antibiotic substitutes. Traditionally, biosafety evaluation of *L. plantarum* relied on phenotypic and toxicological assays, including cell- and animal-based tests for cytotoxicity, allergenicity, and potential pathogenicity [18]. However, these methods are often slow and provide limited insights into complex mechanisms. Advances in next-generation DNA sequencing technologies enable the resolution of complex biological phenomena. By applying genome-mining tools and analyzing gene expression profiles (e.g., virulence genes, ARGs, and metabolic traits), biosafety can be systematically and precisely assessed, providing stronger scientific evidence, in combination with the traditional assays [19,20,21].

The Pacific white shrimp (*Litopenaeus vannamei*) ranks among the world’s most economically important aquaculture species, prized for its excellent nutritional value and strong market demand [22]. However, its perishable nature presents challenges for extending the supply chain and prolonging commercial shelf life, due to its high moisture content (75–84%) and protein content (18–22%) [23]. To combat quality deterioration, chemical preservatives such as sulfites and benzoates have been widely used for decades. Yet, prolonged or improper use of these chemicals raises significant food safety concerns: sulfites can cause allergic reactions and respiratory issues in individuals with asthma [24], while benzoates may react with ascorbic acid to form potentially carcinogenic benzene [25]. Therefore, the development of safer and more effective green preservation technologies is urgently needed [26,27,28]. Among biological preservation methods, lactic acid bacteria (LAB) and their cell-free supernatants (CFS) have attracted considerable attention due to their generally recognized as safe (GRAS) status and diverse antimicrobial metabolites, including organic acids and bacteriocins [29,30]. For example, Nguyen et al. reported that *L. plantarum* T8 and T13 strains inhibited acute hepatopancreatic necrosis disease (AHPND)-causing *Vibrio* species in *L. vannamei*, with a diameter of inhibition zone (DIZ) of 14–18 mm [31]. These findings highlighted considerable promise and underscored the need for further research.

In our previous research, we discovered that the *L. plantarum* D25 strain, isolated from Dongbei kimchi, exhibits tolerance to bile salts, acids and hydrogen peroxide, and has the ability to scavenge 2,2-diphenyl-1-picrylhydrazyl (DPPH) radicals. Additionally, CFS of *L. plantarum* D25 (CFS-*Lp*D25) displayed antimicrobial activity [32]. Building on these findings, this study systematically evaluated antibacterial efficacy, probiotic properties, and biosafety of the *L. plantarum* D25 strain through comprehensive phenotypic and genotypic analyses. The efficacy as a natural biopreservative for refrigerated *L. vannamei* was also evaluated. The results pave the way for the development and application of *L. plantarum* D25 in the food industry.

## 2. Materials and Methods

### 2.1. Bacterial Strain and Culture Conditions

*L. plantarum* D25 was isolated from traditional Dongbei kimchi in China, and identified in our previous research [32]. The strain was routinely cultured in sterile De Man, Rogosa and Sharpe (MRS) medium (pH 6.8, Qingdao Hope Bio-Technology Co., Ltd., Qingdao, China) at 37 °C [32]. The tested bacterial strains (*n* = 18) and corresponding culture media are listed in Table S1 [33].

### 2.2. Antibacterial Activity Assays

The CFS-*Lp*D25 was prepared following the method described by Rao et al. [34]. Briefly, *L. plantarum* D25 was incubated in the MRS medium at 37 °C for 48 h. Subsequently, the culture was centrifuged at 4000 rpm for 10 min at 4 °C. The resulting supernatant was then filtered through sterile 0.22 μm filter membrane (Tiangen Biotech (Beijing) Co., Ltd., Beijing, China), and the filtrate obtained was CFS-*Lp*D25.

The DIZ of CFS-*Lp*D25 against the test bacterial strains (*n* = 18) was measured using the Oxford cup method as described by Chang et al. [35]. Briefly, the sterilized Oxford cups (diameter: 8 mm) were placed on sterile Mueller Hinton Agar (MHA) plates (Thermo Fisher Scientific, Waltham, MA, USA), which had been coated with a bacterial suspension of each tested strain (A_600_ = 0.08–0.13). Absorbance at 600 nm was measured using the BioTek Synergy 2 Multi-Mode Plate Reader (BioTek Instruments, Inc., Burlington, VT, USA). Each Oxford cup contained 150 μL of CFS-*Lp*D25. Streptomycin sulfate (SMS, 100 μg/mL, Sinopharm Chemical Reagent Co., Shanghai, China) solution was used as the positive control (PC), and the MRS medium as the negative control (NC). The MHA plates were incubated at 37 °C for 16–24 h, and DIZ against each tested bacterial strain was then measured, and recorded. In this study, DIZ values greater than 10 mm were interpreted as indicating antibacterial activity, while values greater than 15 mm were considered evidence of a strong antibacterial effect.

The growth inhibition index (GIi) was determined using the liquid growth inhibition method described by Ebrahiminezhad et al. [36] with minor modifications. Briefly, each tested bacterial strain was routinely incubated to mid-logarithmic growth phase (mid-LGP, A_600_ = 0.6–0.8) in trypticase soy broth (TSB) (Beijing Land Bridge Technology Co., Ltd., Beijing, China) (Table S1) at 37 °C. As described above, the prepared CFS-*Lp*D25 (20 μL/well) was added into sterile 96-well plates, and mixed with each tested bacterial culture (80 μL/well). Following a 3 h incubation at 37 °C, 100 μL of fresh TSB (Table S1) was added to each well and incubation continued for an additional 21 h. Subsequently, absorbance at 600 nm of each well was measured using the BioTek Synergy 2 Multi-Mode Plate Reader (BioTek Instruments, USA). Sterile 1 × phosphate-buffered saline (PBS) (Shanghai Sangon Biological Engineering Technology and Service Co., Ltd., Shanghai, China) was used as the control (20 μL/well). The initial CFS-*Lp*D25 was diluted by 10-fold, and a final concentration of 10% CFS-*Lp*D25 was tested in this assay. The GIi value was then calculated as the ratio of the bacterial count in the control group to that in the treatment group (GIi = Nc/Nt × 100%), where Nc and Nt are derived from A_600_ readings of the control and treatment wells, respectively [36].

### 2.3. pH and Organic Acid Assays

The pH of CFS-*Lp*D25 was measured using the FiveGo F2 pH meter (Mettler Toledo, Shanghai, China).

High-performance liquid chromatography (HPLC) was employed to determine concentrations of various organic acids in CFS-*Lp*D25, including ascorbic acid, fumaric acid, glycolic acid, lactic acid, malonic acid, oxalic acid, pyroglutamic acid, propionic acid, succinic acid, and tartaric acid [37]. An Agilent HPLC system (Agilent Technologies, Santa Clara, CA, USA) was equipped with an Agilent ZORBAX SB-Aq column (250 mm × 4.6 mm, 5 μm). The mobile phase consisted of a 40 mmol/L KH_2_PO_4_ solution (Beijing Pharmaceutical Chemical Reagent, Beijing, China) mixed with methanol (Tianjin Concord Technology, Tianjing, China) at a 95:5 volume ratio, with a flow rate of 1.0 mL/min and a column temperature of 30 °C. Standard curves were established, and concentrations of each organic acid were quantified based on their peak area [37].

### 2.4. Analyses of Tolerance of L. plantarum D25 to Stresses

The high-temperature tolerance of *L. plantarum* D25 was examined following the method described by Ma et al. [38] with minor modifications. Briefly, *L. plantarum* D25 was cultured at 37 °C for 24 h. The culture was then centrifuged at 4000 rpm for 10 min. The bacterial pellets were washed twice with the sterile 1 × PBS and resuspended in the PBS to an A_600_ value of 1.0. The suspension was exposed to temperatures of 50 °C, 55 °C, and 63 °C for 1 min, 5 min, 15 min, and 30 min, respectively. Immediately, following heat exposure, the samples were placed in ice, and viable bacterial counts were determined through the agar plate counting method [39].

Additionally, *L. plantarum* D25 was inoculated into the MRS medium, and incubated at 4 °C, 37 °C, and 45 °C for 3 h, 6 h, 12 h, 24 h, and 48 h, respectively. At the specified time points, A_600_ values of each culture were measured and viable bacterial counts were determined [39].

Acid, NaCl, and bile acid tolerance of *L. plantarum* D25 were assessed following the method described by Gokmen et al. [40]. Briefly, *L. plantarum* D25 was inoculated into the MRS medium, which was adjusted to different pH values (3.0 to 6.0) or supplemented with NaCl (0%, 2%, 4%, 5%, 6%, 7%, and 8%), or bile salts (0 mM, 10 mM, and 20 mM) including sodium cholate (NaC), sodium glycocholate (NaGC), and sodium taurocholate (NaTC) (Shanghai Macklin Biochemical Co., Ltd., Shanghai, China), respectively. Growth curves were then measured at 37 °C using the Bioscreen Automated Growth Curve Analyzer (BioTek, USA). The specific growth rate was determined as the slope of the linear regression of the natural logarithm of cell density (A_600_) against time during the exponential growth phase, with the unit of h^−1^.

### 2.5. Carbohydrate Fermentation Assays

The carbohydrate fermentation profile of CFS-*Lp*D25 was determined using the API 50 CHL system (BioMérieux, Marcy l’Etoile, France), according to the manufacturer’s protocol, which tested fermentation of 49 substrates spanning 8 distinct categories, including 15 monosaccharide, 10 sugar alcohol, 7 glycoside, 6 disaccharide, 4 trisaccharide, 3 polysaccharide, 3 sugar acid derivative, and one amino sugar.

### 2.6. Cell Surface Hydrophobicity (CSH), Aggregation Ability, and Biofilm Formation Assays

The CSH of *L. plantarum* D25 was examined using our previously described method [41]. Briefly, an equal volume of 98% hexadecane solution (Sangon, China) was added into the suspension of *L. plantarum* D25 in the 1 × PBS, and the absorbance of the aqueous phase was measured at 600 nm.

Both self-aggregation and co-aggregation capacity assays were performed following the methods by Chen et al. [42]. In brief, *L. plantarum* D25 was cultured in the MRS medium at 37 °C until reaching mid-LGP (A_600_ = 0.6–0.8), and then centrifuged at 4000 rpm for 15 min at 4 °C, washed twice with the sterile 1 × PBS, resuspended in 1 × PBS, and adjusted to an A_600_ of 0.50 ± 0.05. A 4 mL of the bacterial resuspension was vortexed for 10 s and incubated at 37 °C for 2 h. The absorbance at 600 nm was measured from the upper liquid layer. For the co-aggregation assay, the bacterial cell suspension was prepared similarly. Specifically, 2 mL of *L. plantarum* D25 resuspension (A_600_ = 0.50 ± 0.05) was mixed with 2 mL of each tested bacterial strain resuspension (A_600_ = 0.50 ± 0.05), vortexed for 10 s, and incubated at 37 °C for 2 h. A_600_ was measured from the upper liquid layer [42].

*V. cholerae* GIM1.449 was used as an indicator strain in the biofilm formation assay using the crystal violet staining method [43]. Briefly, *V. cholerae* GIM1.449 was cultured in the sterile TSB at 37 °C to the mid-LGP, and the bacterial cells were harvested by centrifugation then suspended with different concentrations of CFS-*Lp*D25 (5%, 10%, 30%, and 100%). CFS-*Lp*D25 was diluted with the fresh TBS, and *V. cholerae* GIM1.449 was adjusted to 10^6^–10^7^ CFU/mL in the suspensions. The TSB and sterial ddH_2_O were used as a negative group and a blank control, respectively. Each suspension (1 mL/well) was inoculated into sterile 24-well cell plates and incubated at 37 °C for 48 h. After incubation, the bacterial culture was discarded, and each well was washed, fixed, air-dried, stained with 0.1% (*w*/*v*) crystal violet for 15–30 min, and then rinsed and dissolved with 95% ethanol (2 mL/well). The absorbance at 595 nm (A_595_) was measured for each well, and the relative biofilm inhibition rate was calculated [43].

### 2.7. Extracellular Polysaccharides (EPSs) Production Assay

The EPSs production was assessed using the Congo red staining method with minor modifications [44]. The MRS medium was supplemented with 3% (*w*/*v*) sucrose and solidified with 1.5% (*w*/*v*) agar. After sterilization and cooling to 55 °C, Congo red dye (Macklin, China) was added to a final concentration of 1 mg/mL, thoroughly mixed, and poured into plates.

Overnight culture of *L. plantarum* D25 were collected by centrifugation, washed with sterile 1 × PBS, and resuspended to A_600_ of 1.0. Then, a 20 µL aliquot of the resuspension was spotted at the center of each Congo red plate, with 1 × PBS serving as a NC. The plates were incubated at 37 °C for 48 h. Colonies exhibiting a dark brown coloration after incubation were recorded as the positive result for EPSs production [44].

### 2.8. Hemolytic and Gelatinase Activity Assays

The hemolytic activity of *L. plantarum* D25 was examined using the method described by Pakshir et al. [45]. *L. plantarum* D25 was inoculated on sterile Columbia blood agar plates (Sangon, China) and incubated at 37 °C for 48 h. Hemolysis was categorized as follows: α-hemolysis, where erythrocytes were partially destroyed, forming a grass-green hemolytic ring around the colony; β-hemolysis, where erythrocytes were completely lysed, resulting in a clear hemolytic ring; and γ-hemolysis, where no change occurred around the colony, indicating intact erythrocytes [45].

The gelatinase activity of *L. plantarum* D25 was assessed following the method by dela Mortezaei et al. [46]. First, 5 mL of sterile gelatin medium (Guangdong Huankai Microbial Sci. & Tech. Co., Ltd., Guangzhou, China) was poured into sterile glass tubes (15 × 150 mm), allowed to solidify. Then, *L. plantarum* D25 was inoculated vertically to the bottom of the tube using a sterile inoculating needle. After incubating at 37 °C for 24 h, the tubes were chilled at 4 °C for 20 min. A lack of solidification upon tilting indicated a positive result for gelatinase activity, whereas solidification indicated a negative result [46].

### 2.9. Antibiotic Susceptibility Assays

The minimum inhibitory concentrations (MICs) of AMP, cefepime (FEP), neomycin sulfate (NEO), hygromycin hydrochloride (HYG), VAN, sodium fusidate (FA) and rifamycin (RIF) (Macklin, China) against *L. plantarum* D25 were assessed using the agar dilution method, following the guidelines of the Clinical and Laboratory Standards Institute (CLSI, M23, 2018), USA [47]. *L. plantarum* D25 was inoculated onto the MRS agar plates containing serial two-fold dilutions of the antibiotics, ranging from 512 to 0.5 μg/mL. The plates were then incubated at 37 °C for 48 h. The MIC was identified as the lowest concentration of the antibiotic that resulted in no visible bacterial growth. *S. aureus* ATCC 25923 served as the quality control strain.

### 2.10. Genome Sequencing, Assembly, and Annotation

The genome of *L. plantarum* D25 was sequenced by Shanghai Majorbio Biomedical Technology Co., Ltd., Shanghai, China using the Illumina Hiseq × 10 platform (Illumina, San Diego, CA, USA). We filtered low-quality sequences and assembled high-quality sequences. Coding sequences (CDS), rRNA genes, and tRNA genes were annotated, and clusters of orthologous groups (COGs) of proteins were predicted, following the methods detailed in our recent reports [48,49].

Genomic islands (GIs), prophages, insertion sequences (ISs), integrons (INTs), clustered regularly interspaced short palindromic repeats (CRISPR)-associated proteins system (CRISPR-Cas systems), virulence-related genes, and ARGs were also predicted using the same procedures described in our recent reports [48,49]. To improve the accuracy of virulence factor prediction, this study applied stricter filtering to the alignment results against the Virulence Factor Database (VFDB, http://www.mgc.ac.cn/VFs/, accessed on 5 March 2025), retaining only matches with both coverage and identity > 70%.

Additionally, plasmid DNA was extracted using the TIANprep Mini Plasmid Kit (TIANGEN Biotech, Beijing, China) following the manufacturer’s protocol. The plasmid pBD33 in *Escherichia coli* was used as a positive control. The extracted samples were analyzed by agarose gel electrophoresis with 1% agarose.

### 2.11. Phylogenetic Tree Construction

A phylogenetic tree was constructed using 52 complete genome sequences of *L. plantarum* retrieved from the GenBank database (Table S2), along with the genome sequence of *L. plantarum* D25 determined in this study, based on 120 core genes analyzed by molecular evolutionary genetics analysis (MEGA, version 10.1.7) software (http://www.megasoftware.net/, accessed on 10 October 2025). To analyze the amino acid dataset of single-copy orthologues from *L. plantarum* genomes, we used OrthoFinder (version 2.5.5) software [50]. A phylogenetic tree was constructed using the FastTree (version 2.1.11) software [51]. We employed the same software parameters as detailed in our recent reports [48,49].

### 2.12. Preservation Applications

The preservation efficacy of CFS-*Lp*D25 on *L. vannamei* was evaluated following established methods [52]. Briefly, fresh *L. vannamei* were collected from Luchao Harbor aquatic product market in Shanghai, China in January, 2026. The shrimp meat samples (1.00 g each) were assigned to six experimental groups: 4 treatment groups treated with CFS-*Lp*D25 at concentrations of 5%, 10%, 30%, or 100% (undiluted CFS-*Lp*D25), respectively; a NC group with distilled water; and a PC group with 0.2% potassium sorbate (Macklin, China) solution. All samples were immersed in corresponding solutions for 10 min, stored at 4 °C, and analyzed at 0 d, 2 d, 4 d, 6 d, and 8 d.

Sensory evaluation: Ten trained evaluators assessed the appearance, color, odor, and other attributes of the samples in an independent laboratory at Shanghai Ocean University (Shanghai, China). A scoring scale from 0 to 5 was used, where a score of 5 indicated excellence, and scores below 3 were deemed unacceptable (Table S3).

pH determination: A 1.00 g aliquot of shrimp meat sample was homogenized with 10 mL of distilled water. Following centrifugation, the pH of the supernatant was measured [52].

Thiobarbituric acid reactive substance (TBARS) determination: A 1.00 g aliquot of the shrimp meat sample was homogenized with a 2.5 mL of distilled water, followed by the addition of 2.5 mL of 10% trichloroacetic acid (Macklin, China). After vortexing and filtration, 0.5 mL of the filtrate was mixed with 0.5 mL of 0.02 mol/L thiobarbituric acid solution (Shanghai Aladdin Biochemical Technology Co., Ltd., Shanghai, China) and heated at 80 °C for 40 min. The absorbance of the mixture was then measured at 532 nm [52].

TVB-N determination: The TVB-N content was determined using an automatic Kjeldahl nitrogen analyzer (Kjeltec^TM^ 9 FOSS Analytical A/S, Hillerød, Denmark) [53].

### 2.13. Inhibitory Activity of CFS-LpD25 Against Vibrio cholerae in Shrimp

The in vitro time-kill assay was performed in liquid broth as described by Liu et al. [53]. Briefly, as described above, *V. cholerae* GIM1.449 culture at mid-LGP was suspended in different concentrations of CFS-*Lp*D25 (5%, 10%, 30%, or 100%) and adjusted to approximately 10^6^–10^7^ CFU/mL. The 0.2% potassium sorbate and fresh TSB were used as a positive control and a negative control, respectively. The suspensions were incubated at 37 °C for 24 h. Samples were collected at 0 h, 3 h, 6 h, 12 h, and 24 h, and viable *V. cholerae* GIM1.449 cells were counted through the agar plate counting method [39].

The shrimp meat samples were prepared according to the method described in our recent reports [54,55]. Briefly, a 10 μL of the *V. cholerae* suspension (A_600_ = 0.6) was inoculated onto the surface of each sample (1.00 g). The experimental groups were set as described in Section 2.12. All the samples were stored at 4 °C. At the specified time points (0 d, 2 d, 4 d, 6 d, and 8 d), each sample was homogenized with 9 mL of sterile 1 × PBS for 3 min, and then subjected to viable bacterial count for bactericidal rate calculation [54,55].

Additionally, the antibacterial effect of CFS-LpD25 on *V. cholerae* GIM1.449 in *L. vannamei* meat was also assessed at different immersion times. The experimental groups were the same as described above. The immersion treatments were performed for 10 min, 20 min, and 30 min. The samples were sealed in sterile homogenization bags and stored at 4 °C. Viable bacterial counts were determined using the plate counting method [39].

### 2.14. Cytotoxicity Assays

The human colon adenocarcinoma cell line Caco-2 (American Type Culture Collection, Manassas, VA, USA) was used in the cell mode. Cytotoxicity was assessed by measuring cell viability using the CCK-8 assay (Dojindo Molecular Technologies, Inc., Rockville, MD, USA) and evaluating cell membrane damage using the lactate dehydrogenase (LDH) release kit (Shanghai Beyotime Biotechnology Co., Ltd., Shanghai, China), following the manufacturers’ instructions. The Caco-2 cells were treated with CFS-*Lp*D25 at final concentrations of 5%, 10%, 30%, or 100%. To eliminate the potential inhibitory effect from the acidity of CFS-*Lp*D25, all treatment solutions were adjusted to pH 7.4 using 1 mol/L NaOH solution prior to use. A 0.2% potassium sorbate solution served as the PC, and MRS medium as the NC.

CCK-8 assay: Briefly, the Caco-2 cells were seeded in sterile 96-well plates containing Dulbecco’s modified eagle medium (DMEM) supplemented with 10% fetal bovine serum (FBS, *v*/*v*) and 1% antibiotic (*v*/*v*, 100 × penicillin-streptomycin concentrate) (Gibco, Waltham, MA, USA) at 37 °C and 5% CO2. The cell culture was then replaced with fresh DMEM medium containing CFS-*Lp*D25 at different concentrations or control solutions. After incubation at 37 °C for 24 h, 10 μL of CCK-8 solution was added to each well, and cells were incubated for an additional 2 h. Absorbance of each well was measured at 450 nm, and relative cell viability was calculated in comparison to the NC group.

LDH release assay: The Caco-2 cell seeding and treatment procedures were identical to those used in the CCK-8 assay. Following the treatment, each Caco-2 cell supernatant was collected and mixed with the reaction substrate according to the instructions of the LDH release kit. Absorbance was measured at 490 nm, and the LDH release rate was calculated to assess the extent of cell membrane damage.

### 2.15. Data Analysis

The SPSS (version 24.0) software (SPSS Inc., Armonk, NY, USA) was utilized to analyze the data using a one-way analysis of variance (ANOVA) and the appropriate post hoc test (Tukey) for multiple comparisons between treatments and controls. Variations were considered statistically significant at *p* < 0.05 [56]. All tests were performed in three independent biological repeats, each of which included technical triplicate.

## 3. Results and Discussion

### 3.1. Antibacterial Profile and Metabolites of CFS-LpD25

#### 3.1.1. Antibacterial Profile of CFS-*Lp*D25

In this study, the antibacterial profile of CFS-*Lp*D25 was determined against 18 bacterial species, including 17 Gram-negative bacteria (e.g., *Enterobacter sakazakii*, *Klebsiella pneumoniae*, *Vibrio alginolyticus*, and *V. cholerae*) and one Gram-positive species (*S. aureus*) (Table 1). The DIZ values ranged from 22.54 mm to 11.44 mm, and GIi values varied from 99.75% to 22.02%. CFS-*Lp*D25 demonstrated strong inhibition (DIZ > 15 mm) against most tested strains (*n* = 12), with particularly potent effects against *V. cholerae* GIM 1.449 and *V. alginolyticus* ATCC 17749, with DIZ values reaching 20.96 mm and 22.54 mm, respectively.

A notable difference was observed in the inhibitory efficacy of CFS-*Lp*D25 against *Vibrio* compared to non-*Vibrio* bacteria. The mean DIZ value against *Vibrio* strains was 17.94 mm with a mean GIi value of 75.20%, whereas for non-*Vibrio* strains, the mean DIZ value was 15.40 mm with a mean GIi value of 67.61%. These results indicated that the antibacterial metabolites produced by *L. plantarum* D25 possibly possess greater specificity or penetrative ability against *Vibrio* species.

The PC (SMS, 100 µg/mL) showed an average DIZ value of 16.54 mm and an average GIi value of 55.75%. Although CFS-*Lp*D25 exhibited a slightly lower average DIZ value (16.15 mm) than SMS, its average GIi value (69.72%) was higher than that of SMS. These results highlighted superior antibacterial activity of probiotic-derived CFS-*Lp*D25 compared to the traditional antibiotic SMS, based on their GIi values.

Considering previous studies, recently, Xie et al. reported that the CFS of *L. plantarum* M616, isolated from sourdough, produced inhibition zones of 14–17 mm against *Escherichia coli*, *S. aureus*, and *Listeria monocytogenes* [57]. In another study, Kim et al. reported that the CFS from *L. plantarum* NIBR97, isolated from Korean kimchi, eliminated 99.9% of *Salmonella* spp. and *E. coli* within 30 min [58].

In summary, CFS-*Lp*D25 demonstrated broad-spectrum antibacterial activity, with strong effects against *Vibrio* species on the basis of the limited number of *Vibrio* species (*n* = 5) tested in this study. It also showed measurable efficacy compared to the antibiotic SMS, underscoring its promising potential as a green biopreservative.

#### 3.1.2. Potential Antibacterial Metabolites in CFS-*Lp*D25

The pH of CFS-*Lp*D25 was measured at 3.83 ± 0.04, which was significantly lower than the tolerance threshold of common pathogenic bacteria (approximately pH 5.5) [59]. Similarly, the low pH has been reported for CFS from other *L. plantarum* strains, such as strains 1A-2 (pH 4.47) and Ca098 (pH 4.46) [60]. This result highlighted the strong acid-producing capacity of *L. plantarum* D25. Such a low pH can directly disrupt cell membrane integrity and inhibit bacterial growth.

As shown in Table 2, the lactic acid content in CFS-*Lp*D25 reached 12.49 mg/mL, greatly exceeding the MIC of lactic acid against *S. aureus* ATCC 25923 (MIC = 1.28 mg/mL) [61]. This high lactic acid concentration contributed to the broad-spectrum inhibitory effect of CFS-*Lp*D25. Additionally, the presence of tartaric acid (2.83 mg/mL) and succinic acid (2.62 mg/mL) in CFS-*Lp*D25 could disrupt bacterial energy metabolism by chelating metal ions (such as Mg^2+^/Fe^2+^) or competitively inhibiting key enzymes in the tricarboxylic acid (TCA) cycle (such as α-ketoglutarate dehydrogenase) [62]. Malonic acid (2.21 mg/mL) in CFS-*Lp*D25, a specific inhibitor of succinate dehydrogenase, could further disrupt the electron transport chain in bacterial cells, thereby affecting cellular energy metabolism. To provide experimental evidence for these suggested antibacterial mechanisms, enzyme activity, membrane permeability, or microscopy assays should be further carried out in future research.

Collectively, based on these results, we hypothesized that CFS-*Lp*D25 establishes an effective antibacterial foundation through multiple molecular mechanisms: low pH disrupts bacterial cell membrane structure; high concentrations of lactic acid, tartaric acid, succinic acid, and malonic acid induce intracellular acidification and inhibit key enzymes in central metabolic pathways; and malonic acid interferes with energy metabolism. Together, these components created a multi-target, synergistic antibacterial network mediated by CFS-*Lp*D25.

### 3.2. Probiotic Characteristics of L. plantarum D25

#### 3.2.1. Tolerance of *L. plantarum* D25 to Different Temperatures

**Figure 1:**
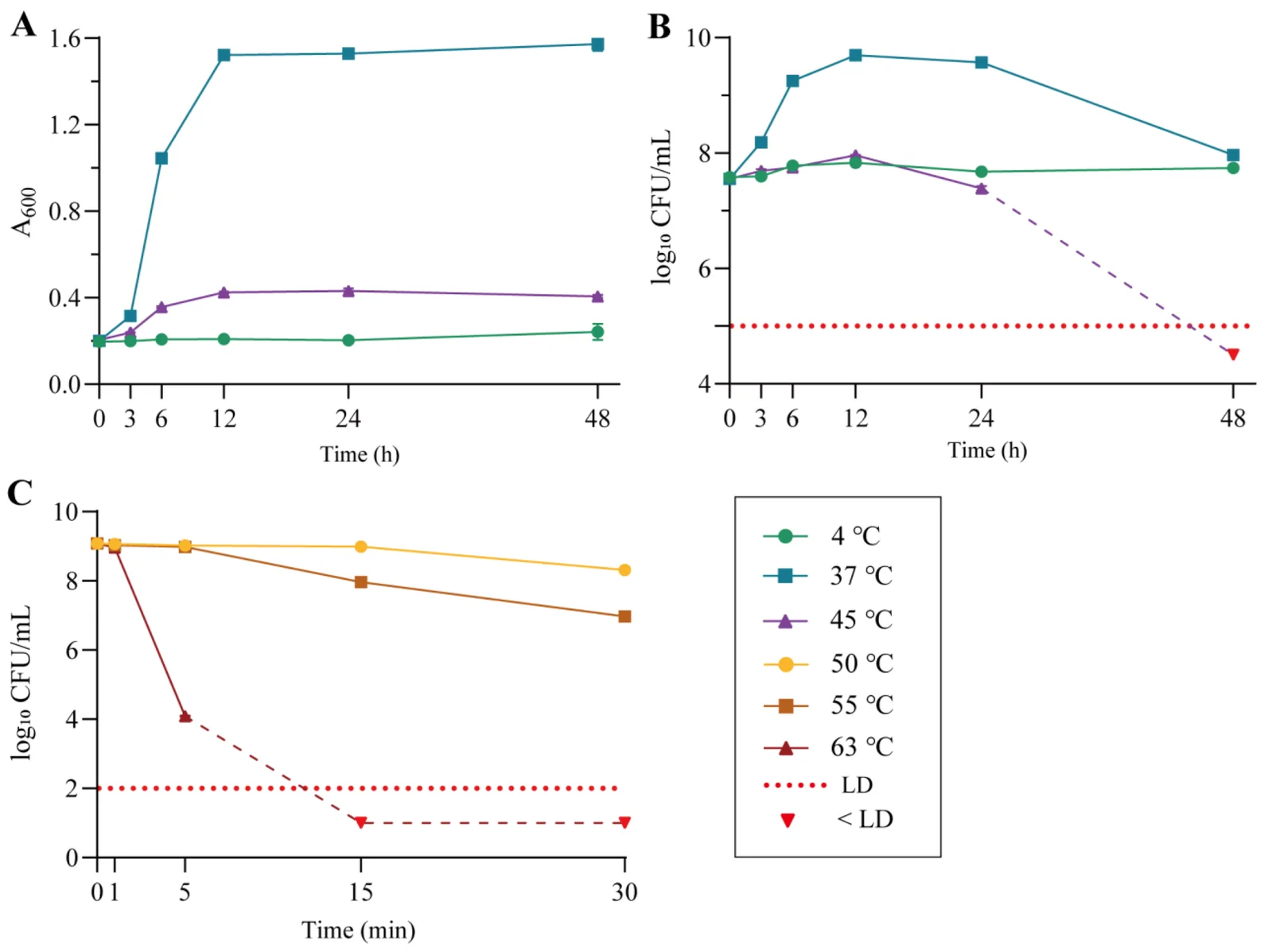
Tolerance of *L. plantarum* D25 to different temperatures. (**A**) Growth curves of *L. plantarum* D25 incubated in the MRS at 4 °C, 37 °C, and 45 °C for 48 h. (**B**) Viable cell counts of *L. plantarum* D25 incubated in the MRS at 4 °C, 37 °C, and 45 °C for different times. The red dashed line indicates the limit of detection (10^5^ CFU/mL); ▼ denotes data points below the detection limit (no viable cells detected). (**C**) Inactivation curves of *L. plantarum* D25 under heat treatments. *L. plantarum* D25 at mid-LGP (A_600_ = 0.6–0.8) were collected, washed, and resuspended in 1 × PBS, and then subjected to heat treatments at 50 °C, 55 °C, and 63 °C for 0–30 min. The red dashed line marks the limit of detection (10^2^ CFU/mL).

As shown in Figure 1A,B, *L. plantarum* D25 exhibited pronounced temperature-dependent growth across a range of low to moderate temperatures (4 °C to 45 °C). Its growth kinetics were optimal at 37 °C: A_600_ values increased from 0.201 to 1.049 within 6 h, and viable cell counts peaked at 12 h (9.70 log_10_ colony forming unit (CFU)/mL). At 4 °C, *L. plantarum* D25 entered metabolic dormancy, with viable cell counts remaining stable over 48 h, confirming strong cold-storage stability. After exposure to 45 °C for 24 h, the bacterial survival rate remained 69.81%, but fell below the detection limit (5 log_10_ CFU/mL, survival < 0.28%) after 48 h, indicating good tolerance to short-term moderate heat (≤45 °C, 24 h) but severe inactivation after prolonged stress (45 °C, 48 h), possibly due to cumulative cellular damage.

The thermal inactivation assay assessed the heat sensitivity of *L. plantarum* D25 (Figure 1C). The strain showed notable tolerance at 50–55 °C: after treatment at 50 °C or 55 °C for 30 min, viable cell counts fell to 8.31 log_10_ CFU/mL and 6.97 log_10_ CFU/mL, respectively. The D_50_ (39 min) and D_55_ (14.2 min) were substantially higher than those reported for *L. plantarum* CIDCA 83114 (D_55_ = 7.50 min) [63]. Notably, *L. plantarum* D25 was highly sensitive at 63 °C: complete inactivation (<10^4^ CFU/mL) occurred within 5 min, and the D_63_ value was approximately 1 min—much shorter than the typical D_63_ range for *Lacticaseibacillus casei* (4.7–7.3 min).

Collectively, *L. plantarum* D25 demonstrated strong heat resistance at 50–55 °C (D_50_ = 39 min, D_55_ = 14.2 min) but underwent rapid inactivation at 63 °C (D_63_ ≈ 1 min), offering distinct advantages for industrial use. First, its compatibility with moderate processing allows high viable cell counts to be maintained during pasteurization or fermentation at ≤55 °C (after 55 °C for 30 min, residual counts exceeded 6.97 log_10_ CFU/mL), reducing the cost of encapsulation techniques. Second, a brief heat treatment at 63 °C (≤5 min) can serve as a natural “biological switch” to precisely terminate fermentation, preventing overacidification while preserving cell integrity for postbiotic applications. Third, compared with *Lactobacillus casei* under typical inactivation conditions (70 °C, 15 min), its rapid high-temperature inactivation can reduce sterilization energy consumption by more than 35%. This dual thermal-sensitivity profile of *L. plantarum* D25 thus balances product safety with production efficiency.

#### 3.2.2. Tolerance of *L. plantarum* D25 to Acid, Osmotic and Bile Salt Stresses

**Figure 2:**
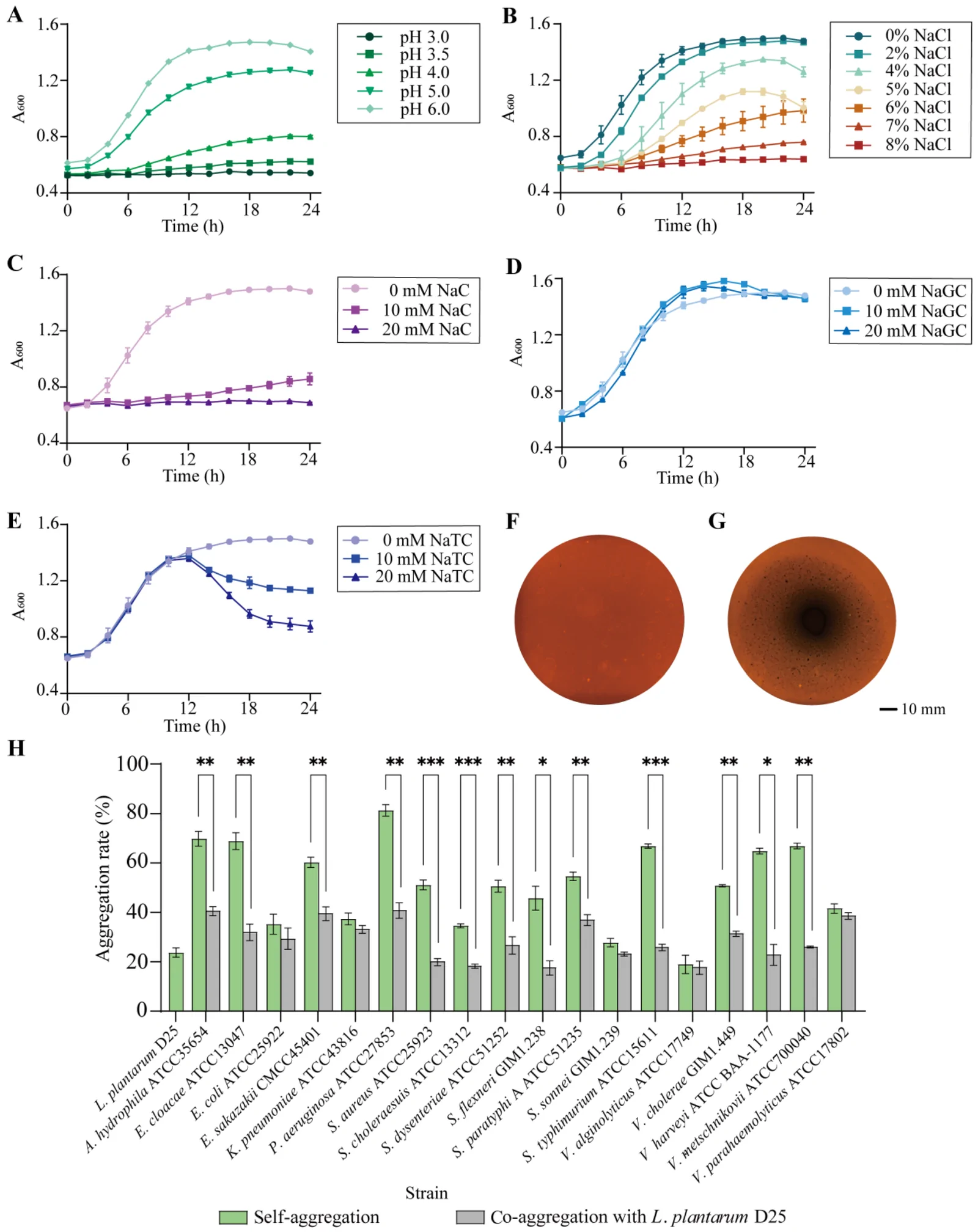
Probiotic characteristics of *L. plantarum* D25. (**A**–**E**) Tolerance of *L. plantarum* D25 to acid (pH 3.0–6.0), osmotic pressure (0–8% NaCl), and bile salts including NaC (0–20 mM), NaGC (0–20 mM), and NaTC (0–20 mM). (**F**,**G**) Congo red staining assay for EPSs production: the NC showing no color change; and *L. plantarum* D25, exhibiting a dark brown color for the positive EPS production. (**H**) Self aggregation rates of 18 bacterial species and co-aggregation rates with *L. plantarum* D25. *, *p* < 0.05; **, *p* < 0.01; and ***, *p* < 0.001.

The effectiveness of probiotics relies on their ability to survive and colonize the host gastrointestinal tract [64]. For probiotics to confer health benefits, they must endure the harsh acidity of the stomach and remain viable in the small intestine, which contains bile salts and undergoes osmotic fluctuations. To assess these characteristics, we measured the growth curves of *L. plantarum* D25 under various stress conditions, including acidity (pH 3 to 6), NaCl (0% to 8%), and bile salts including NaC (0–20 mM), NaGC (0–20 mM), and NaTC (0–20 mM) (Figure 2A–E).

Figure 2A shows the growth dynamics of *L. plantarum* D25 across a pH range of 3.0 to 6.0. The strain achieved optimal growth at pH 5.5–6.0, with a brief lag phase (3.5 h) and a high specific growth rate (0.104 h^−1^). At pH 4.0, the lag phase was significantly extended to 8.5–5 h longer than the control group (pH 6.0). Nevertheless, the strain successfully entered the LGP and proliferated, reaching a maximum biomass (A_600_ = 0.804) at 21.5 h, indicating strong acid tolerance. The strain’s ability to survive under highly acidic conditions underscores its potential as a robust oral probiotic candidate.

As shown in Figure 2B, the growth of *L. plantarum* D25 was progressively inhibited as NaCl concentrations increased from 2% to 8%. At 2% NaCl, the lag phase extended to 5 h compared to the control group (3.5 h, 0% NaCl), yet the strain still reached a high final biomass (A_600_ = 1.470), suggesting effective osmotolerance. At 6% NaCl, growth was severely hindered, with maximum biomass decreasing by 60.56% relative to the control. These findings suggested that *L. plantarum* D25 is suitable for use in fermented foods with moderate salt concentrations (≤4%).

As illustrated in Figure 2C, *L. plantarum* D25 was completely inhibited under high NaC (20 mM) conditions. However, at 10 mM NaC, the strain showed growth with a maximum biomass of A_600_ = 0.186. Since NaC concentrations in the human intestine generally range from 0.7 to 7 mM [65], *L. plantarum* D25 may be able to survive or remain viable while exposed to bile salts during gastrointestinal transit. NaGC showed no harm to the growth of *L. plantarum* D25. At 10 mM NaGC, the maximum biomass (A_600_ = 1.583) slightly exceeded that of the control group (A_600_ = 1.501, 0% NaGC) (Figure 2D). In Figure 2E, *L. plantarum* D25 also demonstrated substantial tolerance to NaTC (<20 mM). At 10 mM and 20 mM NaTC, maximum biomass decreased to A_600_ = 1.381 and A_600_ = 1.364, reductions of 7.99% and 9.13%, respectively, versus the control. Previous studies have shown that *L. plantarum* D25 exhibits in vitro bile salt hydrolase (BSH) activity [32]. Collectively, these findings suggested that *L. plantarum* D25 possibly contributed to cholesterol reduction.

In summary, these results indicated that *L. plantarum* D25 likely withstands simulated gastrointestinal (10 mM NaC, 10 mM NaGC, and <20 mM NaTC) and food processing stresses (pH 3.0 to 6.0, and ≤4% NaCl), providing foundational evidence for its probiotic properties and highlighting potential directions for future research and industrial application.

#### 3.2.3. Carbohydrate Fermentation and EPS-Producing Capabilities of *L. plantarum* D25

As shown in Table 3, *L. plantarum* D25 demonstrated a robust metabolic capacity for a wide range of carbohydrates. It efficiently utilized monosaccharides such as glucose, fructose, and galactose, as well as disaccharides and oligosaccharides including sucrose, lactose, and raffinose. By rapidly consuming available sugars and producing acids, it established a notable competitive advantage. Importantly, *L. plantarum* D25 also metabolized plant secondary metabolites such as amygdalin, arbutin, and aesculin, indicating its ability to degrade polyphenolic derivatives, likely due to its adaptation in a plant-based ecological niche. Additionally, its positive metabolic response to trehalose is significant, a carbohydrate fermentation profile that may serve as an auxiliary characteristic for its phenotypic identification [66].

However, this strain was unable to utilize starch and inulin, as confirmed by genomic analyses showing the absence of key genes involved in their metabolism, such as *amyA*, *glgP*, *malEFG*, *inu*, *fosE*, and *fruA*. This limitation significantly restricts its potential application in whole grain fermentation or dietary fiber conversion. Additionally, *L. plantarum* D25 showed negative responses to xylose and its isomers, as well as to glycerol and erythritol, suggesting poor growth in environments with high concentrations of polyols. As a result, it may struggle to effectively utilize lignocellulosic hydrolysates as a carbon source.

In summary, *L. plantarum* D25 exhibited excellent metabolic performance on substrates rich in monosaccharides and plant-derived compounds, making it well-suited for fruit and vegetable fermentation systems. However, its metabolic limitations with complex polysaccharides and polyols constrain its application in cellulose-based substrates. To overcome these challenges, approaches such as genetic modification or co-cultivation may be required to enhance its metabolic adaptability.

Additionally, as illustrated in Figure 2F,G, after incubation on MRS agar plates containing Congo red and sucrose for 48 h, the colonies of *L. plantarum* D25 displayed a dark brown color, indicating positive EPSs activity. This characteristic was further supported by genomic analyses showing that glycosyltransferase genes (*L. plantarum* D25*_1112*, *_1113*, *_1135*, and *_1136*) were present in its GIs (GI 03), similar to other *L. plantarum* strains involved in EPSs biosynthesis [67]. The production of EPSs is a key functional trait of probiotics, contributing to their colonization in the intestinal environment and their adaptation to food matrices [44].

#### 3.2.4. CSH and Aggregation Ability of *L. plantarum* D25

The CSH of *L. plantarum* D25 was measured at 21.22%. Although this represents a moderately low level, it still supports essential surface adhesion functions [68]. The self-aggregation rate of *L. plantarum* D25 was 23.52%, a moderate range (20% to 40%), indicating that the strain is less likely to form large, and dense aggregates. This property may facilitate more uniform distribution throughout the intestinal tract rather than confinement to specific regions [69]. Additionally, this moderate self-aggregation suggested a balanced adhesion capability, which may help prevent excessive colonization or unintended immune responses.

As shown in Figure 2H, self-aggregation rates among the tested bacteria (*n* = 18) varied broadly from 18.75% to 81.02%. Thirteen strains exhibited high self-aggregation (>40%), with *Pseudomonas aeruginosa* ATCC 27853 demonstrating the highest rate (81.02%). Other notably high self-aggregators included *Aeromonas hydrophila* ATCC 35654 (69.54%), *Enterobacter cloacae* ATCC 13047 (68.63%), *Vibrio metschnikovii* ATCC 700040 (66.63%), and several others. The remaining four species displayed moderate self-aggregation (20% to 40%), e.g., *E. coli* ATCC 25922 (35.03%) and *K. pneumoniae* ATCC 43816 (37.12%). *V. alginolyticus* ATCC 17749 had the lowest rate at 18.75% (<20%).

It is noteworthy that the co-aggregation ability of the tested bacteria with *L. plantarum* D25 ranged from 17.70% to 40.48%. With the exception of *V. parahaemolyticus* ATCC 17802 (38.54%), *K. pneumoniae* ATCC 43816 (33.18%), *E. coli* ATCC 25922 (29.18%), *S. sonnei* GIM1.239 (23.20%), and *V. alginolyticus* ATCC 17749 (17.70%), the co-aggregation ability of the remaining 13 strains with *L. plantarum* D25 was significantly lower than their self-aggregation rates (*p* < 0.05). The comparison demonstrated that these 13 strains tended to self-aggregate more readily than to associate with *L. plantarum* D25. This observation suggested a unique probiotic mechanism: although *L. plantarum* D25 exhibited relatively low co-aggregation with these strains, such interactions may disrupt their self-aggregation, thereby inhibiting their biofilm formation and virulence expression [70].

To provide experimental evidence for the suggested mechanism, we further investigated the impact of CFS-*Lp*D25 on biofilm formation of *V. cholerae* GIM1.449 (Figure S1). The results showed that CFS-*Lp*D25 effectively inhibited the bacterial biofilm formation in a dose-dependent manner. The inhibition rate was 67.6% for 5% CFS-*Lp*D25, increased to 86.9% for 10% CFS-*Lp*D25, and reached a maximum of 96.9% for 30% CFS-*Lp*D25. The 100% CFS-*Lp*D25 treatment yielded a slightly lower inhibition rate of 93.4% than 30% CFS-*Lp*D25. These results confirmed that CFS-*Lp*D25 can effectively reduce biofilms of *V. cholerae* GIM1.449.

In summary, the probiotic traits of *L. plantarum* D25 were evident in its sophisticated balance strategy. Its moderate self-aggregation and adhesion properties possibly enable effective colonization and distribution within the host intestine, providing a foundation for its beneficial effects. Furthermore, its moderate co-aggregation ability with pathogenic bacteria may act as an interference mechanism, leading to suppress their self-aggregation behaviors.

### 3.3. Biosafety Assessment of L. plantarum D25

#### 3.3.1. Hemolytic and Gelatinase Activity of *L. plantarum* D25

Hemolytic activity is a key safety indicator used to assess the potential pathogenicity of *L. plantarum* [71]. Gelatinase activity reflects bacterial proteolytic capability. In this study, *L. plantarum* D25 exhibited neither hemolytic nor gelatinase activity (Figure S2), supporting its safety and reliability for use in food and medicine to help regulating host intestinal microecology.

#### 3.3.2. Antibiotic Susceptibility of *L. plantarum* D25

The MICs of five classes of antibiotics against *L. plantarum* D25 were assessed: β-lactams (AMP), aminoglycosides (KAN, NEO, HYG), glycopeptides (VAN), rifamycins (RIF), and fusidic acid (FA). *L. plantarum* D25 was phenotypically sensitive to AMP but resistant to FA, RIF, KAN, NEO, HYG, and VAN. The quality control strain *S. aureus* ATCC 25923 showed resistance only to HYG (MIC = 128 μg/mL) and was sensitive to the other tested antibiotics (Table 4).

Specifically, the MIC value for AMP was 2 μg/mL, meeting the European Food Safety Authority (EFSA) safety threshold (≤2 μg/mL) and confirming the strain’s sensitivity and safety. In contrast, KAN displayed a high MIC value of 512 μg/mL, far exceeding the EFSA breakpoint (≤64 μg/mL), which indicates a significant risk and potential transfer of ARGs. VAN also showed an MIC of 512 μg/mL, which is considered an intrinsic resistance trait of the *Lactiplantibacillus* genus and does not pose a safety threat as it is not transmissible [72,73]. For FA (MIC = 32 μg/mL), RIF (MIC = 8 μg/mL), NEO (MIC = 256 μg/mL), and HYG (MIC = 512 μg/mL), EFSA has not established breakpoints. Compared to *S. aureus* ATCC 25923, these MIC values were significantly higher and therefore classified as resistant.

In summary, *L. plantarum* D25 was resistant to FA, RIF, KAN, NEO, VAN, and HYG. However, VAN resistance is an inherent characteristic of the genus and not considered a safety risk, which was validated by whole-genome sequencing and analysis (see below).

#### 3.3.3. Genome Features of *L. plantarum* D25

Whole-genome sequencing enables the identification of key biosafety risk factors, such as ARGs, virulence factors, and MGEs [74]. Therefore, in this study, we determined the complete genome sequence of *L. plantarum* D25. The strain contained no plasmid, as the extraction of plasmids yielded no small plasmid DNA (Figure S3), and BLAST (version 2.3.0) analysis revealed no large plasmids in the genome of the strain either.

**Figure 3:**
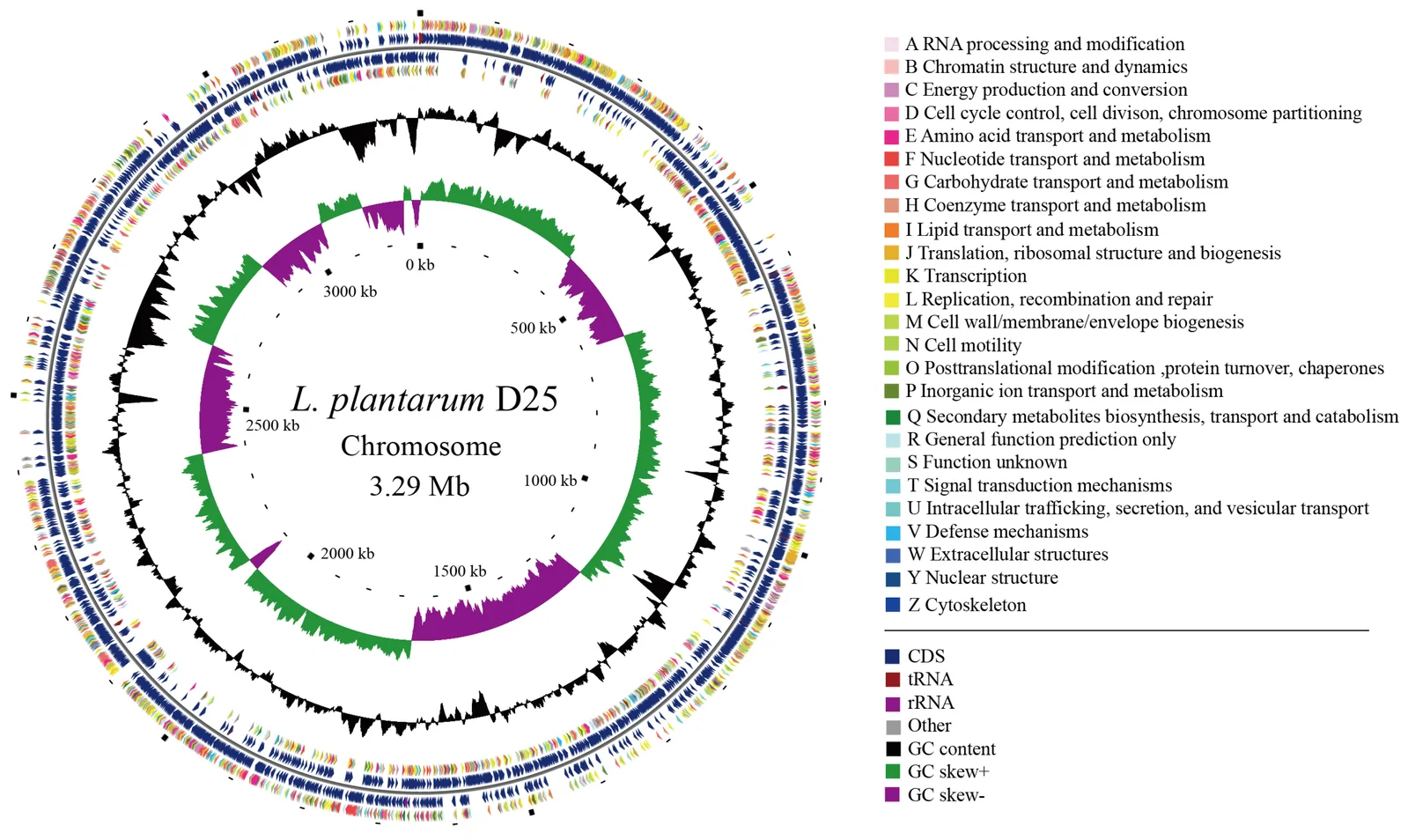
Genome characterization and phylogenetic relationship of *L. plantarum* D25. Circular genome map of *L. plantarum* D25. From the inner to outer circles: genome scale (100 kb intervals); GC skew (green and purple indicate positive and negative values, respectively); GC content (protrusions indicate values above the mean, and recessions below); annotation of CDS, tRNA, and rRNA genes on the negative-strand; gene function on the negative-strand, colored according to the COG function categories; annotation of CDS, tRNA, and rRNA genes on the positive strand; and gene function on the positive-strand, colored according to the COG function categories.

A total of 8,362,486 high-quality sequencing reads were obtained, resulting in the assembly of 17 scaffold sequences with an average sequencing depth of 387.51 × fold. The assembled genome was 3,290,333 bp in size, with a GC content of 44.44%. A total of 3,077 coding sequences (CDS) were predicted, with 79.72% assigned to functional categories in the COG database (Figure 3; Table S4).

##### Phylogenetic Relatedness of *L. plantarum* D25

A phylogenetic tree was constructed using whole-genome sequences of 52 *L. plantarum* strains retrieved from the GenBank database (Table S2; Figure S4). These strains were chosen on the basis of diverse environments and geographical origins between 2010 and 2025, involving human samples (feces, intestines, jejunum, dental abscesses; *n* = 11), an animal sample (chicken; *n* = 1), various foods (pickles, sourdough, preserved products, sour bamboo shoots, fermented tofu, wine, grapes; *n* = 39), and other sources (*n* = 1). Their geographical origins spanned Asia (China, India, South Korea, and Turkey), Europe (France, and Italy), North America (United States), and South America (Argentina).

This analysis grouped the 53 genomes into six major clusters (Group 1 to Group 6). Notably, *L. plantarum* D25 (GenBank assembly No. GCA_055502195.1) was clustered in Group 1, forming a highly supported sister branch with *L. plantarum* CNW10 (GenBank assembly No. GCF_001633285.1), a strain isolated from the adult intestinal environment in the United States in 2013. This suggested that these two strains shared a recent common ancestor and are closely related genetically and evolutionarily. This relationship provides opportunities for practical applications of *L. plantarum* D25 in medicine or biotechnology, depending on the context of its sister strain of human intestinal origin.

##### Possible MGEs in *L. plantarum* D25 Genome

The dual role of MGEs necessitates their careful evaluation in the biosafety assessment of *L. plantarum*. While vigilance is required regarding the risks of HGT of virulence factors and ARGs, it is also important to recognize the evolutionary benefits MGEs confer, such as enhanced stress tolerance and EPSs biosynthesis.

GIs are among the most significant HGT elements, often carrying genes with crucial biological functions. In this study, eight GIs (GI 01 to GI 08) were identified in the genome of *L. plantarum* D25, ranging from 4.6 to 46.8 Kb in length (Figure S5; Table S5). These GIs contained 7 to 55 predicted genes that encoded proteins with diverse biological roles.

For example, GI 03 (46.8 Kb), located on Scaffold 2, is one of the largest GIs in *L. plantarum* D25. This region contains several glycosyltransferase family 2 proteins (*L. plantarum* D25*_1112*, *_1113*, *_1135*, and *_1136*), which are responsible for assembling the EPSs backbone by linking activated sugar units [75]. GI 03 also encodes oligosaccharide flipase family proteins (*L. plantarum* D25*_1136*) involved in transporting synthesized oligosaccharide units across the cell membrane. Overall, GI 03 may contribute essential probiotic functions by synthesizing EPSs that could enhance gut colonization, immune modulation, and biofilm formation, based on the in silico data.

Additionally, GI 07 encodes a sophisticated cell wall regulatory hub, featuring a two-component signaling system with the response regulator YycF (*L. plantarum* D25*_2082*) and the cell wall metabolism sensor histidine kinase WalK (*L. plantarum* D25*_2083*) [76]. This system is crucial for maintaining cell wall integrity and homeostasis, allowing the strain to better withstand harsh intestinal conditions.

Gene function prediction of the GIs in *L. plantarum* D25 revealed a complex genetic structure that balances probiotic functions and safety considerations. GIs such as GI 03, GI 05, and GI 06 comprise the core probiotic modules, while GI 01, GI 07, and GI 08 enhance adaptability through phage resistance, cell wall stress response, and survival in extreme environments. However, these elements may also pose safety risks related to genomic instability and prophage induction. The probiotic potential of *L. plantarum* D25 arises from the synergistic effects of these diverse GIs, highlighting the importance of comprehensive risk assessment in strain development.

Phages are among the most abundant biological entities on Earth and play crucial roles in biogeochemical cycling, HGT, and shaping microbial community composition [77]. Three prophages (Ph 01 to Ph 03) were identified in the *L. plantarum* D25 genome, ranging from 11.1 to 40.3 Kb and encoding 15–52 predicted genes (Figure S5; Table S6). Ph 02 lacked a complete set of structural phage proteins and contained no virulence factors or ARGs, indicating negligible biosafety risk. Ph 01 and Ph 03 possessed core genes required for the lytic cycle, representing temperate phages, but neither harbored virulence factors or ARGs, indicating low risk. The closest relatives of Ph 01 and Ph 02 were PHAGE_Lactob_Sha1 (NC_019489, 41.7 kb), a temperate phage in the *Siphoviridae* family infecting *Lactobacillus* species and commonly found in fermented food. Genome analysis found no hazardous elements, such as toxins or ARGs, and its presence does not significantly impact host safety [78]. The closest relative of Ph 03 was PHAGE_Lister_B025 (NC_009812, 42.7 kb) [79], another temperate *Siphoviridae* phage targeting *Listeria* species. It has been reported that this phage has been used in biocontrol of foodborne pathogens, with experimental validation showing no virulence and successful passage of preliminary inflammation and toxicity tests [80]. In summary, the predicted prophages in the *L. plantarum* D25 genome do not contain any known risk factors, and their homologous phages have favorable safety profiles in both natural and industrial environments. Thus, the presence of these prophages does not adversely affect the biosafety of *L. plantarum* D25.

The CRISPR–Cas system is an important adaptive immune mechanism in prokaryotes, enabling recognition and degradation of invading genetic elements such as phages and plasmids [80]. The genome of *L. plantarum* D25 lacks this system, indicating a possible vulnerability: the strain may lack a critical defense against HGT of ARGs and virulence determinants in the environment.

ISs are highly efficient vectors for HGT, capturing and expressing ARGs and increasing the risk of MDR [81]. No ISs were detected in the genome of *L. plantarum* D25, indicating a reduced risk of acquiring and spreading ARGs. Additionally, no INTs were identified either.

Collectively, these results demonstrated a low risk of ARG transmission, supporting the biosafety of *L. plantarum* D25 for use as a potential probiotic or food starter culture, although we could not rule out a possible risk raised by the missing of CRISPR-Cas system.

##### Predicted Potential Virulence Determinants in *L. plantarum* D25 Genome

In the *L. plantarum* D25 genome, three gene sequences showed high similarity to entries in the VFDB: elongation factor EF-Tu (*L. plantarum* D25*_0111*), hyaluronan capsule synthase (*L. plantarum* D25*_0748*), and ATP-dependent Clp protease proteolytic subunit (*L. plantarum* D25*_0778*) (Table S7).

EF-Tu is a highly conserved translation elongation factor responsible for delivering aminoacyl-tRNAs during protein synthesis. In *L. plantarum* CS242, EF-Tu has been identified as a multifunctional protein supporting colonization and survival in harsh environments (e.g., the gastrointestinal tract) [82]. In non-pathogenic bacteria, EF-Tu’s multifunctionality is not associated with virulence but instead contributes to proper protein folding and adaptation to environmental stresses [83].

While the hyaluronic acid capsule acts as a key virulence factor in *Streptococcus pyogenes*, homologous genes in *L. plantarum* serve markedly different functions. Evidence has indicated that capsule-associated genes in *L. plantarum* are typically involved in the synthesis of general EPSs, rather than hyaluronic acid [84]. These products primarily facilitate bacterial adhesion, biofilm formation, and immune modulation [84].

ClpP is an ATP-dependent protease involved in protein degradation and stress responses [85]. Although ClpP can regulate virulence factors in pathogens like *Listeria*, its main role in non-pathogenic bacteria is maintaining protein homeostasis rather than promoting pathogenicity. For instance, in *L. plantarum* LRCC5310, ClpP is associated with acid and bile tolerance, aiding adaptation to the host environment [86].

In summary, VFDB annotations are largely derived from pathogen studies, which can result in homologous sequences being misclassified as “virulence factors” but overlook their beneficial functions in non-pathogenic bacteria. The three so-called virulence factors annotated in the *L. plantarum* D25 genome do not pose a safety risk; instead, they contribute to the strain’s probiotic properties.

##### ARGs in *L. plantarum* D25 Genome

Eight ARGs (*L. plantarum* D25*_0111*, *_0389*, *_0976*, *_0977*, *_0979*, *_0981*, *_0982*, and *_2032*) were predicted in the *L. plantarum* D25 genome (Table S8). Previous research has suggested that these genes commonly arise from point mutations and represent intrinsic or mutationally acquired resistance, not horizontal acquisition from external sources [87]. In this study, the genomic analysis also revealed no MGEs flanking these genes.

Clinically, the antibiotics targeted by these ARGs are primarily non-first-line or clinically restricted agents. For instance, aminocoumarins linked to *gyrB* and *parE* have been withdrawn from widespread use due to toxicity concerns, and the intrinsic resistance of *Lactobacilli* to drugs such as RIF and STP has been classified by the EFSA as low risk and not a safety concern.

The genomic analysis of *L. plantarum* D25 revealed that ARGs (*parE*, *gyrB*, *rpoB*, and *rpsL*) were chromosomally encoded and not associated with MGEs, reflecting the strain’s phenotypic profile. For example, although a high MIC for KAN (512 μg/mL) was observed, this finding is consistent with a previous report suggesting that low cell wall permeability may contribute to its intrinsic resistance to KAN [88]. Moreover, their chromosomal location limits HGT. Similarly, the MIC for RIF (8 μg/mL) was explained by *rpoB* mutations, and the MIC for STP (512 μg/mL) was correlated with *rpsL* [89,90]. The genomic analyses confirmed these resistance traits were intrinsic or mutationally acquired [88,89,90], reinforcing that the risk of HGT from *L. plantarum* D25 was negligible. Taken together, these findings indicated that the AR profile of *L. plantarum* D25 meets probiotic safety evaluation criteria.

### 3.4. Time-Kill Kinetics In Vitro and Application of CFS-LpD25 in Shrimp Preservation Under Refrigeration

#### 3.4.1. Time-Kill Kinetics of CFS-*Lp*D25 on *V. cholerae* in Liquid Broth

**Figure 4:**
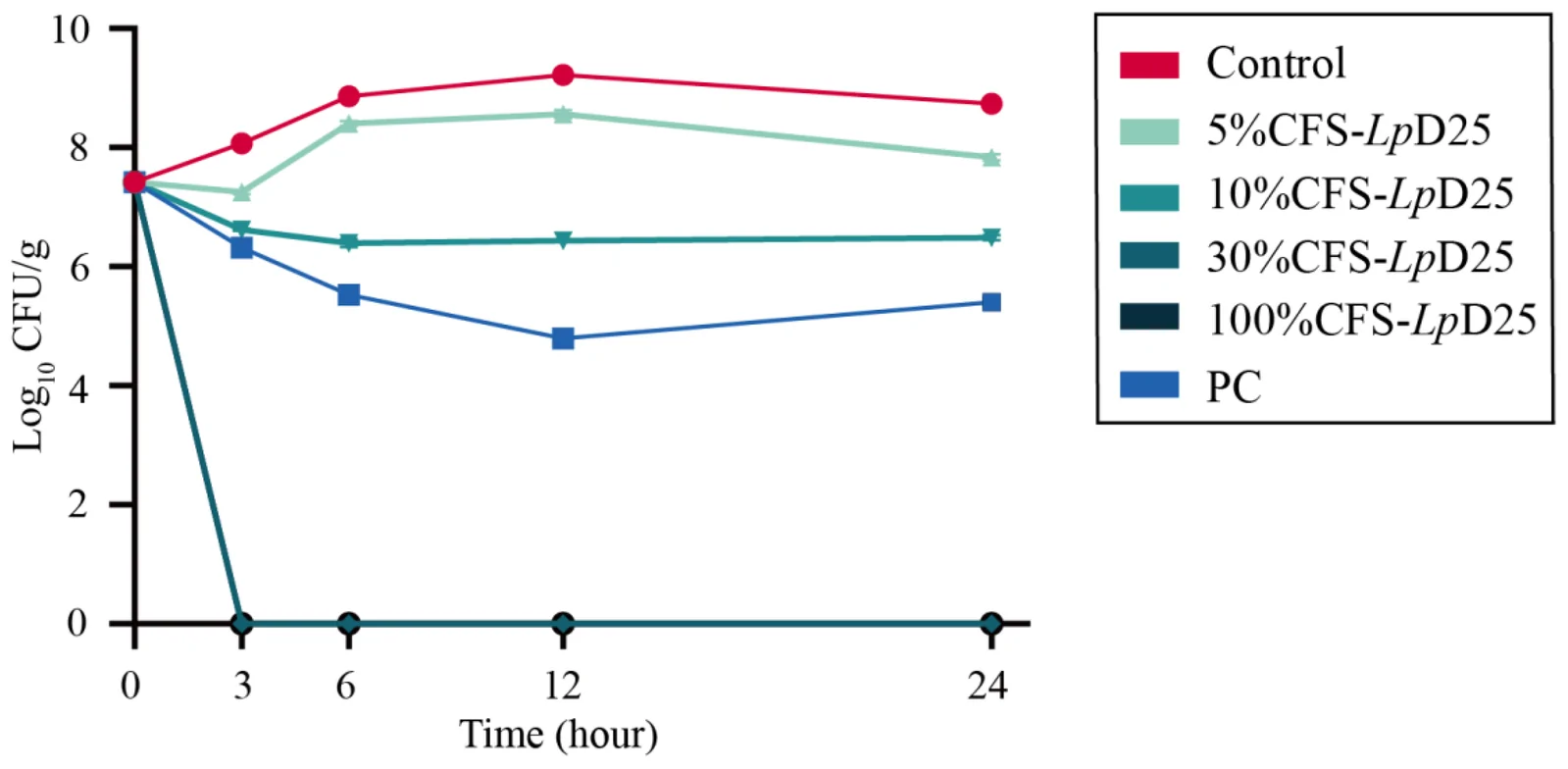
Time-kill kinetics of *V. cholerae* GIM1.449 in the TSB exposed to gradient concentrations of CFS-*Lp*D25. Time-kill kinetics curves triggered by 30% CSF-*Lp*D25 and 100% CSF-*Lp*D25 completely overlapped.

As shown in Figure 4, in the untreated group, viable counts of *V. cholerae* GIM1.449 were 7.42 log_10_ CFU/mL at 0 h, reached a peak of 9.22 log_10_ CFU/mL at 12 h, and declined to 7.57 log_10_ CFU/mL at 24 h, when incubated in the TSB. In the treatment groups, 5% CFS-*Lp*D25 mildly inhibited the bacterial growth with a 0.66 log_10_ reduction at 12 h compared with the untreated control; the treatment with 10% CFS-*Lp*D25 exerted substantially enhanced antibacterial activity, with viable counts maintained at approximately 6.4 log_10_ CFU/mL from 6 h to 24 h, and the overall efficacy was comparable to that of the positive control (0.2% potassium sorbate).

Remarkably, 30% and 100% CFS-*Lp*D25 exhibited rapid and irreversible bactericidal effects: viable cells dropped below the limit of detection (<10 CFU/mL) within 3 h of exposure, and no regrowth was observed during the entire 24 h incubation period. These results demonstrated a concentration-dependent transition from bacteriostatic to bactericidal action of CFS-*Lp*D25.

#### 3.4.2. Antibacterial Efficacy of CFS-*Lp*D25 on *V. cholerae* in *L. vannamei*

##### Initial Antibacterial Activity

**Figure 5:**
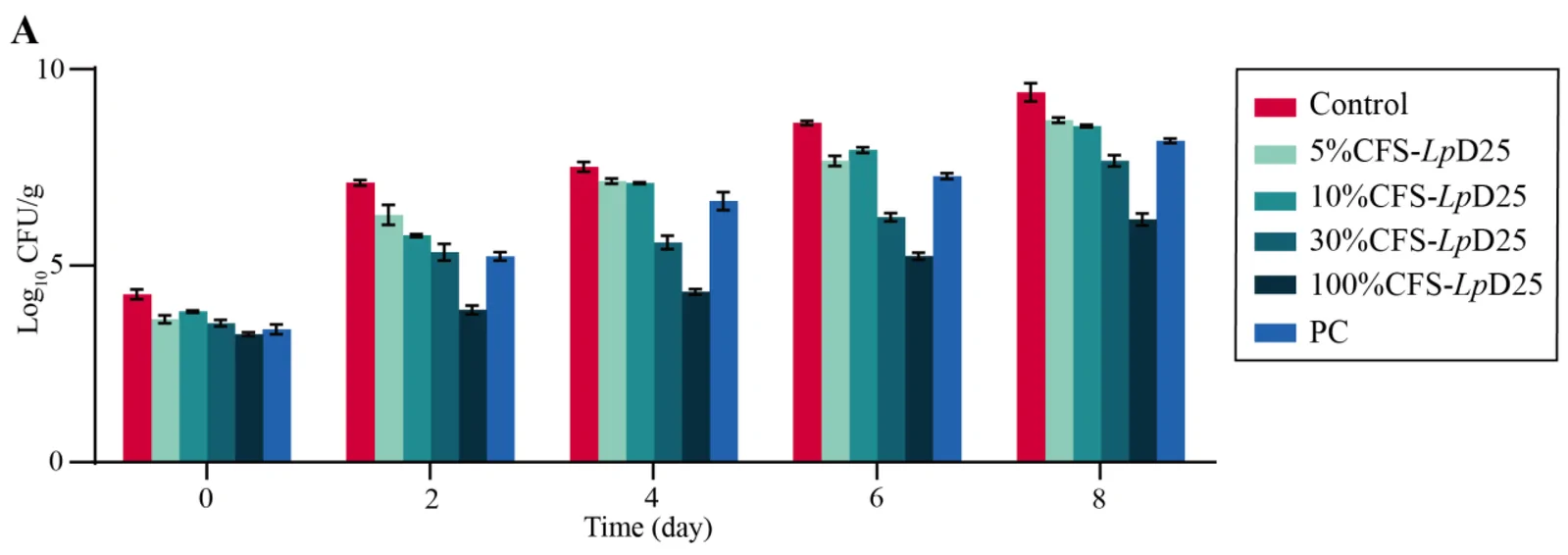
Changes in viable counts of *V. cholerae* GIM1.449 in *L. vannamei* meat samples after treated with CFS-*Lp*D25 for 8 days at 4 °C. The treatment groups contained 5%, 10%, 30%, or 100% CFS-*Lp*D25. PC: Treated with 0.2% potassium sorbate. Control: Untreated with fresh TSB.

*V. cholerae* is a common food-borne pathogen that can cause severe cholera in humans worldwide. This bacterium is frequently isolated from aquatic products, including shrimp [91]. As shown in Figure 5, the untreated control group showed no reduction in viable count of *V. cholerae*, whereas the PC group (0.2% potassium sorbate) exhibited an immediate inhibition effect, with the initial cell count decreasing from 4.27 log_10_ CFU/g to 3.28 log_10_ CFU/g—a reduction of 87.07% at day 0.

The CFS-*Lp*D25-treated groups also demonstrated initial antibacterial activity, with reductions in viable count of *V. cholerae* ranging from 63.95% to 90.44% depending on the concentrations (5–100%). Notably, the 30% CFS-*Lp*D25 group achieved the same antibacterial effect as the PC group during this initial phase. This immediate reduction is critical for decontaminating the product surface following post-harvest contamination and lowering the initial microbial load before storage.

##### Prolonged Antibacterial Activity

As shown in Figure 5, the growth dynamics of *V. cholerae* changed markedly during 8 days of refrigerated shrimp storage. In the untreated control group, the bacterial viable count rose sharply to 9.41 log_10_ CFU/g on day 8. In contrast, the PC group showed a decrease to 8.18 log_10_ CFU/g, representing a 94.67% reduction. These findings indicated that antibacterial efficacy of potassium sorbate was limited during the storage condition.

In contrast, the CFS-*Lp*D25-treated groups demonstrated superior long-term antibacterial activity against *V. cholerae*. The antibacterial rates for the 30% and 100% CFS-*Lp*D25 treatment groups reached 98.30% and 99.95% on day 8, respectively, significantly surpassing potassium sorbate. This superior performance can be attributed to the synergistic action of compounds present in CFS-*Lp*D25. The high levels of lactic acid (12.49 mg/mL) established an environment inhospitable to the *V. cholerae* growth, while organic acids disrupted bacterial cell membranes and interfered with metabolic pathways.

A limitation of the “soaking method” was observed in the potassium sorbate group and the CFS-*Lp*D25-treated groups, as both treatments proved insufficient to prevent pathogen regrowth over prolonged storage.

##### Antibacterial Effect of CFS-*LpD25* in *L. vannamei* Meat at Different Immersion Times

To screen an appropriate immersion process that balances antibacterial efficacy and sensory quality of *L. vannamei* meat, three immersion durations (10 min, 20 min, or 30 min) were set to investigate inhibitory effects of different concentrations of CFS-*Lp*D25 against *V. cholerae* in shrimp meat.

The initial load of *V. cholerae* on shrimp meat in the untreated control group was 4.21 ± 0.04 log_10_ CFU/g, and no significant fluctuation in viable counts was observed within 30 min, indicating that the shrimp matrix itself had no significant effect on the bacterial survival.

**Figure 6:**
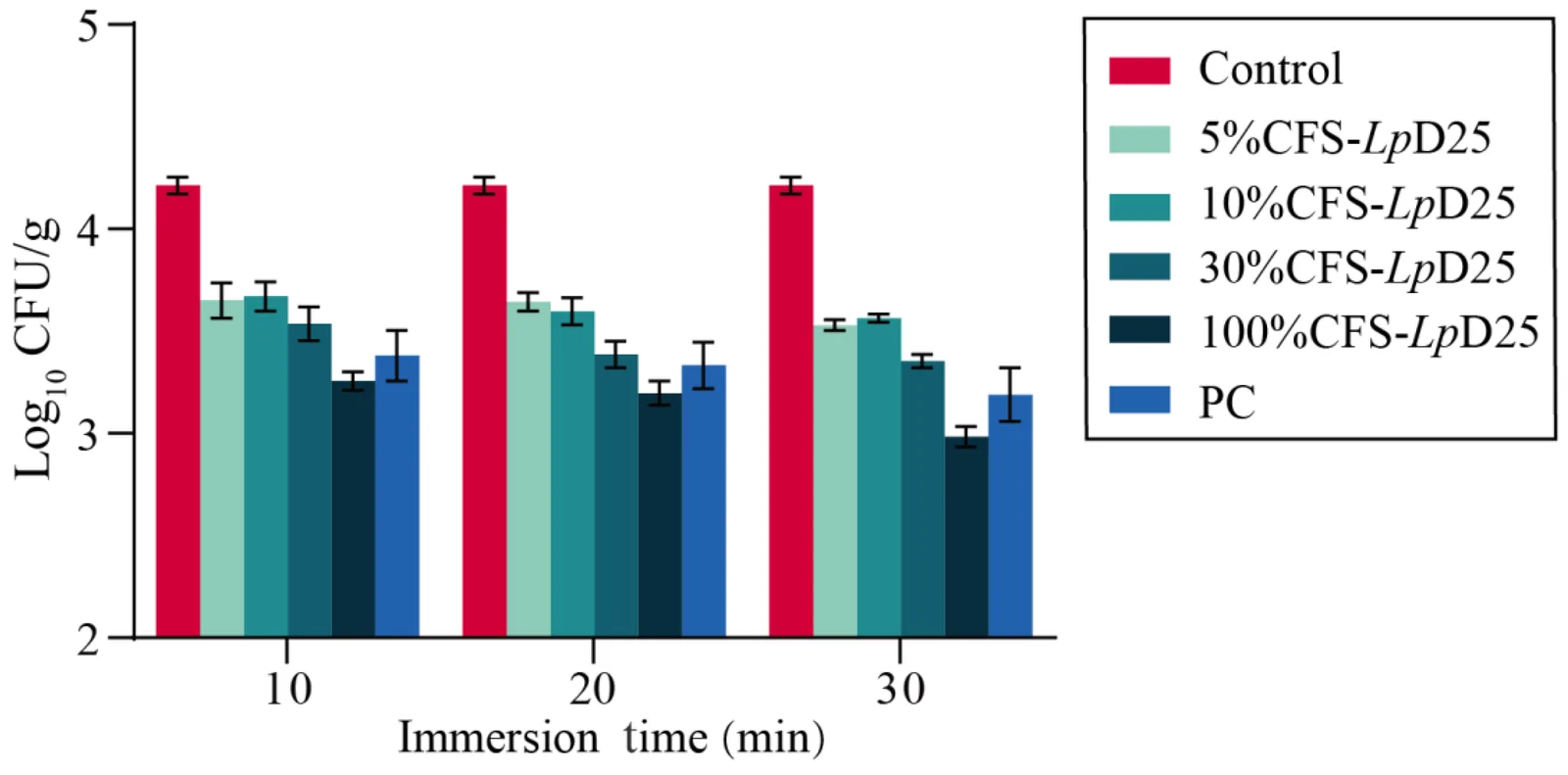
Antibacterial effect of CFS-*Lp*D25 against *V. cholerae* GIM1.449 in *L. vannamei* meat at different immersion times.

As shown in Figure 6, the antibacterial effect of CFS-*Lp*D25 increased slightly with prolonged immersion time at all concentrations, but the additional antibacterial gain from extended immersion was very limited. For instance, in the low-concentration 5% CFS-*Lp*D25 group, when immersion time was extended from 10 min to 30 min, viable counts only decreased by 0.12 log_10_ CFU/g, showing the weak time-dependent enhancement; in the 10% CFS-*Lp*D25 group, 10 min of immersion reduced bacterial counts by 0.54 log_10_ CFU/g compared with the control; extending immersion to 30 min only increased the inhibition amplitude by 0.11 Log_10_ CFU/g. In the high-concentration 30% CFS-*Lp*D25 group, 10 min of immersion resulted in a 0.67-log reduction; when extended to 30 min, the inhibition amplitude increased by 0.19 log_10_ CFU/g, with a total reduction of 0.86 log_10_ CFU/g, which was comparable to the effect of the 0.2% potassium sorbate positive control. In the 100% CFS-*Lp*D25 group, 10 min of immersion achieved a 0.95-log reduction, and the antibacterial effect was superior to that of 0.2% potassium sorbate; further extending immersion to 20 min and 30 min only provided additional reductions of 0.06 and 0.28 log_10_ CFU/g, respectively, indicating a low marginal benefit of extending the exposure time.

From the perspective of product quality, CFS-*Lp*D25 is an acidic fermentation system. Prolonged immersion could aggravate acid denaturation of shrimp myofibrillar proteins, which may lead to deterioration of sensory quality such as texture softening, color lightening, and decreased elasticity. In contrast, 10 min short-term immersion has less impact on the texture, color, and flavor of shrimp meat, which better meets the quality control requirements of aquatic product processing.

In conclusion, extending immersion time has no significant improvement on the antibacterial effect of CFS-*Lp*D25. Considering both antibacterial efficacy and retention of product sensory quality, 10 min is preferentially recommended as the process time for CFS-*Lp*D25 immersion treatment.

#### 3.4.3. Preservative Efficacy of CFS-*Lp*D25 on *L. vannamei*

##### Sensory Quality Evaluation

**Figure 7:**
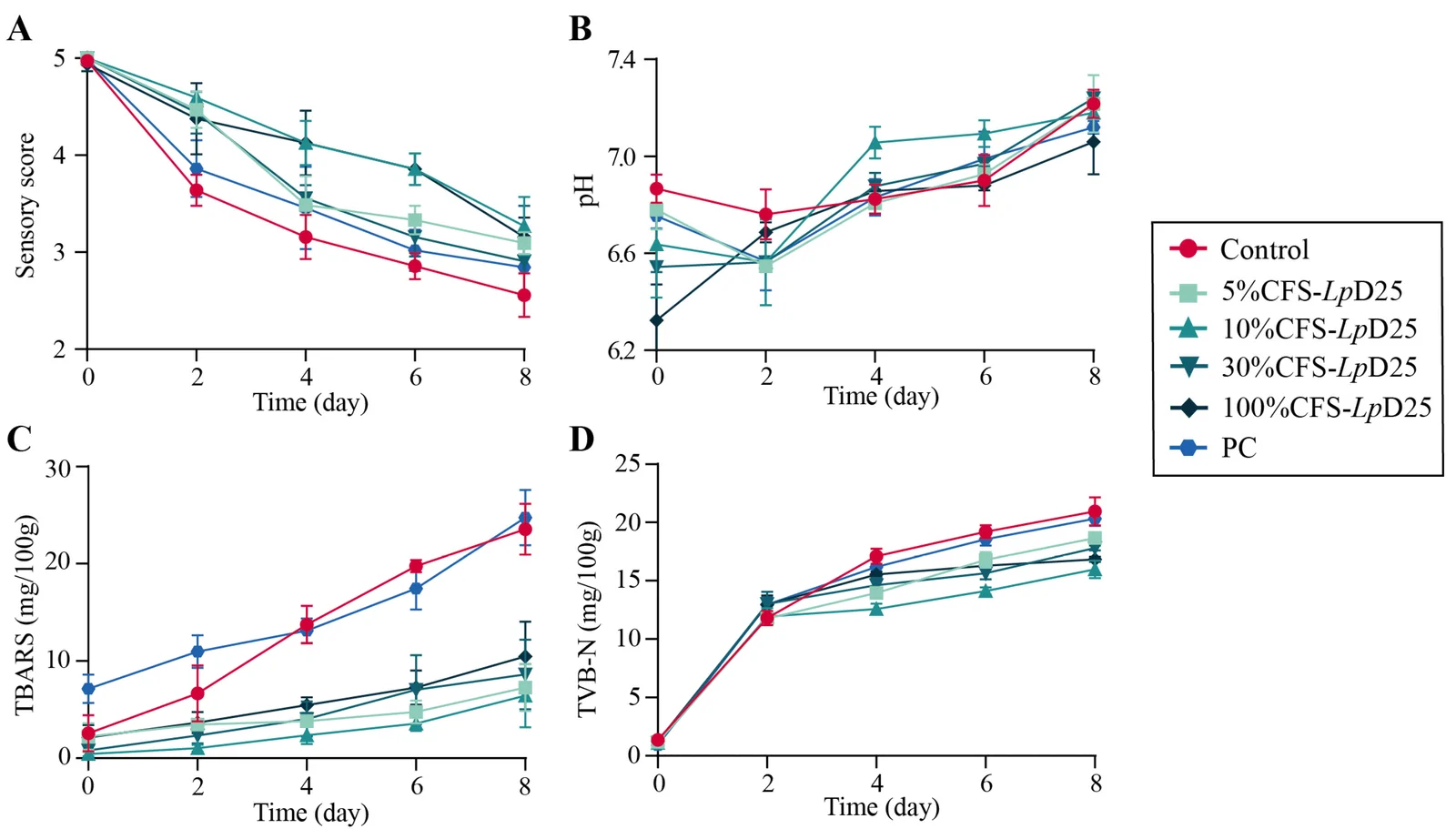
Changes in *L. vannamei* meat samples in sensory evaluation scores (**A**), pH (**B**), TBARS (**C**), and TVB-N (**D**) values after treatment with CFS-*Lp*D25 (5–100% concentrations) for 8 days at 4 °C.

Sensory attributes are critical determinants of consumer acceptance. As shown in Figure 7A, the untreated control group exhibited the fastest deterioration, with sensory score (score: 2.85) dropping below the acceptable threshold by day 6 during storage at 4 °C. This rapid decline could possibly be attributed to endogenous enzyme activity and accelerated microbial growth, which produced off-odors and softened texture.

The PC group (0.2% potassium sorbate) showed only a slight delay in sensory decline compared to the control, with scores falling below the acceptability threshold on day 8 (score: 2.84). By contrast, the CFS-*Lp*D25 treatment groups (5–100%) exhibited notably better sensory retention. On day 8, the 5%, 10%, and 100% CFS-*Lp*D25 groups maintained sensory scores above the acceptable threshold (3.09, 3.27, and 3.39, respectively). Statistical analysis confirmed that the 10% and 100% CFS-*Lp*D25-treated groups significantly inverted sensory quality decline compared to the untreated control (*p* < 0.01).

An interesting anomaly emerged regarding the concentration effects of CFS-*Lp*D25. Higher concentrations generally led to enhanced antibacterial results; however, it is important to note that higher concentrations of CFS-*Lp*D25 may pose biosafety risks (see the following cytotoxicity of CFS-*Lp*D25). At 30% and 100% concentrations, the acidity induced protein denaturation on the shrimp surface, resulting in flocculent precipitates and undesirable texture. The 30% concentration appeared to be a “transition zone” where acid-induced damage was most pronounced.

##### pH Dynamics

The pH value is a dynamic indicator of post-mortem glycolysis and spoilage. As shown in Figure 7B, the untreated control group followed the classic crustacean pattern [92]: an initial pH drop from lactic acid accumulation (such as anaerobic glycolysis), followed by a steady increase as alkaline compounds (e.g., ammonia and trimethylamine) formed via amino acid degradation.

All CFS-*Lp*D25-treated groups showed an initial pH decline proportional to concentrations, reflecting the acidic nature of CFS-*Lp*D25 (pH 3.83). During the storage, a divergence in pH trends emerged. Most CFS-*Lp*D25 treatment groups followed the classic “drop-then-rise” curve, but the 100% CFS-*Lp*D25 group exhibited a monotonic increase in pH from day 0. This suggested that the high CFS-*Lp*D25 concentration fully suppressed glycolysis, eliminating the early pH drop.

Additionally, CFS-*Lp*D25-treated groups displayed a more rapid pH increase during the later storage compared to the untreated control.

##### Lipid Oxidation

Lipid oxidation is a primary driver of quality deterioration in seafood, causing rancidity and color changes [93]. The TBARS value quantifies secondary oxidation products, mainly malondialdehyde (MDA). As shown in Figure 7C, TBARS values increased in all groups during the storage at 4 °C.

Importantly, TBARS increase was significantly lower in CFS-*Lp*D25-treated groups than in the untreated control and PS groups. For example, while the untreated control, and PS groups showed steep TBARS rises (2.60 to 23.54 mg/100 g, and 7.15 to 24.74 mg/100 g), the 10%, and 30% CFS-*Lp*D25-treated groups maintained much lower TBARS (0.44 to 6.43 mg/100 g, and 0.80 to 8.58 mg/100 g), indicating strong antioxidant capacity. This effect was likely multifactorial: the acidic environment and possible bioactive peptides in CFS-*Lp*D25 may chelate pro-oxidant metal ions (such as irons from hemoglobin) or scavenge free radicals, thereby slowing the oxidation chain reaction in *L. vannamei*.

##### Protein Degradation

TVB-N is a key chemical indicator of freshness, reflecting protein degradation by endogenous enzymes and spoilage microbes. As shown in Figure 7D, the untreated control group exhibited a rapid increase in TVB-N value, indicating advanced spoilage, whereas all treatment groups had significantly lower increases (*p* < 0.05). On day 8, the 10% CFS-*Lp*D25-treated group achieved the lowest TVB-N (15.96 mg/100 g) among all groups, significantly lower than both the untreated control (20.94 mg/100 g) and PC (20.33 mg/100 g) groups (*p* < 0.01). These results highlighted the effectiveness of CFS-*Lp*D25—especially at 10% concentration—in suppressing proteolytic activity and deaminases. Maintaining low TVB-N levels was critical for extending the shrimp shelf life, aligning with the sensory evaluation results.

#### 3.4.4. Cytotoxicity of CFS-*Lp*D25

Cytotoxicity assessment using the Caco-2 cell line took advantage of its established utility for simulating intestinal barrier function in vitro [94], making it a relevant model for evaluating the potential effect of CFS-*Lp*D25 upon the human intestinal tract. Cellular metabolic status was evaluated with the CCK-8 assay, which measures mitochondrial activity, while membrane integrity was assessed by the LDH release assay [11]. The combination of these approaches provided a comprehensive assessment of Caco-2 cell viability, with outcomes classified as non-cytotoxic (cell viability ≥ 70%), mildly cytotoxic (cell viability between 50% and 70%), or significantly cytotoxic (cell viability < 50%) [95].

**Figure 8:**
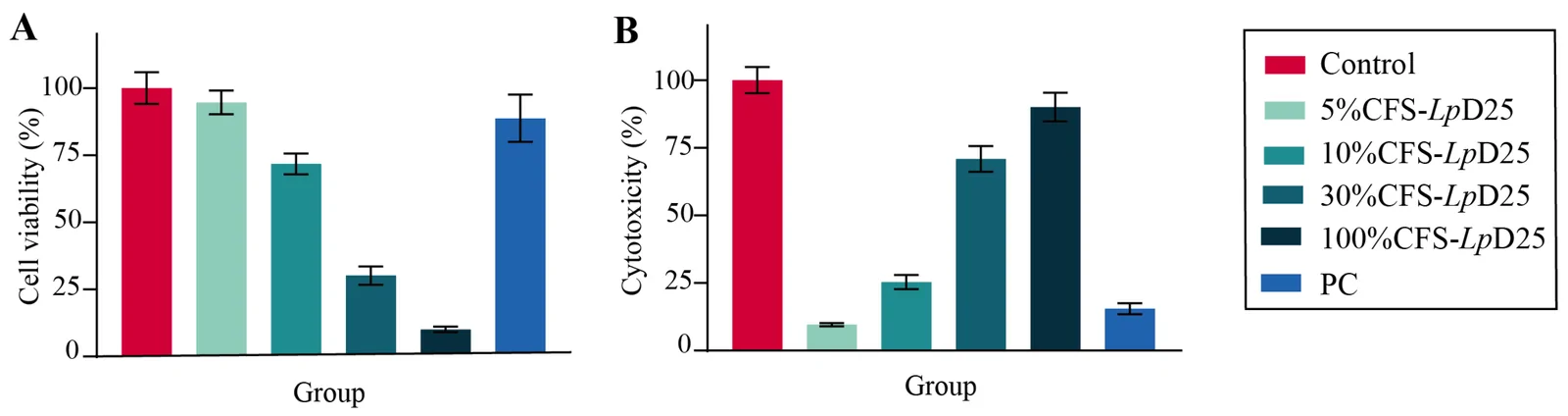
Cell viability and membrane integrity of Caco-2 cells assessed by the CCK-8 (**A**), and LHD release (**B**) assays.

As shown in Figure 8A,B, the cytotoxicity of CFS-*Lp*D25 toward Caco-2 cells was concentration-dependent. The 5% and 10% CFS-*Lp*D25 treatment groups exhibited non-cytotoxic effects, with 94.69% and 71.78% viability of Caco-2 cells, respectively, whereas the 30% (30.19% viability) and 100% (10.07% viability) CFS-*Lp*D25-treated groups showed significant cytotoxicity. The LDH release assay results supported these observations: the 30% and 100% CFS-*Lp*D25 groups demonstrated marked cell destruction, with 70.86% and 90.03% LDH release, respectively. Notably, the PC group’s performance was similar to the 5% CFS-*Lp*D25 group, suggesting that CFS-*Lp*D25 at low concentrations caused irritation comparable to the conventional preservative in the intestinal tract.

The model cell used in this study could not grow normally under acidic conditions when supplemented with CFS-*Lp*D25; therefore, pH-neutralization was necessary. As a result, the findings with pH-neutralized CFS*-Lp*D25 may not accurately represent the effects of the native form to which Caco-2 cells were exposed. The observed inhibition of CFS-*Lp*D25 on Caco-2 cells at neutral pH may contribute to unidentified small molecules, or osmotic pressure effects, which should be further investigated in future research. The potential risks can be effectively mitigated in practical use through appropriate post-treatment or dosage control of CFS-*Lp*D25.

## 4. Conclusions

*L. plantarum* D25 demonstrated probiotic potential. CFS-*Lp*D25 exhibited broad-spectrum antibacterial activity against 17 bacterial species, with particularly strong inhibition effects on *V. cholerae* GIM 1.449 and *V. alginolyticus* ATCC 17749, with DIZ values reaching 20.96 mm and 22.54 mm, respectively. This activity was driven by multifactorial mechanisms involving low pH and high concentrations of organic acids, especially lactic acid (12.49 mg/mL). The strain showed resilience to simulated gastrointestinal stresses, maintaining growth at low pH (3.83), in the presence of NaCl (≤4%), and tolerating bile salt concentrations up to 20 mM. It also displayed adhesion properties, including moderate autoaggregation (23.52%), cell-surface hydrophobicity (21.22%), and significant co-aggregation with various pathogens.

*L. plantarum* D25 exhibited biosafety characteristics. Phenotypic assays confirmed the absence of hemolytic and gelatinase activities, ruling out basic toxicological risks. Genomic analysis revealed no virulence determinants. While phenotypic resistance to several antibiotics (such as KAN and VAN) was observed, genomic analysis revealed that these ARGs were located on the chromosome and not associated with MGEs, indicating a low risk of HGT.

*L. plantarum* D25 demonstrated significant potential for industrial application. It exhibited notable dual thermal sensitivity, tolerating temperatures of 50–55 °C, but being rapidly inactivated at 63 °C, which enables precise industrial process control. Carbohydrate fermentation profiling indicated efficient utilization of various mono- and disaccharides, but limited ability to degrade complex polysaccharides (such as starch and inulin). This trait can inform targeted industrial applications.

Importantly, this study validated the practical application of *L. plantarum* D25 as a potential natural biopreservative. In refrigerated *L. vannamei*, treatment with 10% CFS-*Lp*D25 extended shelf life by effectively controlling sensory deterioration, pH changes, lipid oxidation (TBARS), and protein degradation (TVB-N), outperforming the chemical preservative potassium sorbate. Additionally, CFS-*Lp*D25 inhibited the proliferation of *V. cholerae* in the refrigerated *L. vannamei* meat. Cytotoxicity assessment using the human intestinal Caco-2 cell line confirmed that CFS-*Lp*D25 was non-cytotoxic at effective concentrations (5–10%), supporting its biosafety for food applications. Its long-term biosafety should be further investigated through in vitro and in vivo animal mode experiments in future research.

In summary, the integrated phenotypic and genotypic analyses demonstrated that *L. plantarum* D25 is a potential probiotic candidate. Its strong antimicrobial activity and gastrointestinal tolerance as a natural biopreservative position it as a promising strain for reducing reliance on chemical preservatives in the food industry and advancing the development of functional foods.

## Figures and Tables

**Table 1:** Antibacterial profile of CFS-*Lp*D25 against 18 species of bacteria.

Strain	*L. plantarum* D25		PC	
	DIZ (mm)	GIi (%)	DIZ (mm)	GIi (%)
*Aeromonas hydrophila* ATCC 35654	12.79 ± 1.19 *	31.75 ± 1.86 *	15.34 ± 0.98	42.85 ± 4.71
*Enterobacter cloacae* ATCC 13047	15.32 ± 1.28 ***	28.34 ± 1.84 ***	-	10.10 ± 3.23
*Enterobacter sakazakii* CMCC 45401	16.40 ± 1.08	98.10 ± 0.15	16.55 ± 1.82	96.19 ± 2.87
*Escherichia coli* ATCC 25922	14.97 ± 0.91 *	98.46 ± 0.13 ***	16.98 ± 0.40	27.06 ± 6.14
*Klebsiella pneumoniae* ATCC 43816	16.81 ± 2.15 *	97.44 ± 0.24 *	21.79 ± 0.52	98.26 ± 0.34
*Pseudomonas aeruginosa* ATCC 27853	-	22.02 ± 1.56 *	14.26 ± 0.04	34.82 ± 5.93
*Salmonella choleraesuis* ATCC 13312	11.44 ± 0.56 ***	97.69 ± 0.31	14.00 ± 0.38	95.51 ± 2.60
*Salmonella paratyphi* ATCC 51235	17.41 ± 2.00	28.93 ± 3.83 **	17.03 ± 1.38	14.69 ± 2.90
*Salmonella typhimurium* ATCC 15611	14.33 ± 2.10 *	94.69 ± 1.32	18.91 ± 1.34	93.20 ± 1.40
*Shigella dysenteriae* ATCC 51252	17.07 ± 0.69	32.91 ± 1.60 ***	15.76 ± 1.60	98.40 ± 0.99
*Shigella flexneri* GIM 1.238	18.69 ± 2.44	95.25 ± 0.85	17.47 ± 1.48	91.68 ± 2.83
*Shigella sonnei* GIM 1.239	13.88 ± 2.39	55.33 ± 0.97	17.03 ± 4.98	50.33 ± 42.67
*Staphylococcus aureus* ATCC 25923	15.64 ± 0.33	98.02 ± 0.20 ***	16.03 ± 0.24	98.02 ± 0.20
*Vibrio alginolyticus* ATCC 17749	22.54 ± 0.95 ***	73.57 ± 1.47 ***	13.01 ± 0.38	12.32 ± 3.17
*Vibrio cholerae* GIM 1.449	20.96 ± 0.58 **	98.68 ± 0.31 ***	17.95 ± 0.37	24.36 ± 1.40
*Vibrio harveyi* ATCC BAA-1177	15.22 ± 1.38	66.35 ± 2.32 ***	16.59 ± 1.04	13.06 ± 1.40
*Vibrio metschnikovii* ATCC 700040	16.48 ± 1.15 *	99.75 ± 0.08	18.95 ± 0.67	91.87 ± 6.81
*Vibrio parahaemolyticus* ATCC 17802	14.52 ± 0.26	37.65 ± 2.15 ***	13.52 ± 0.60	10.78 ± 1.77

Note: *, significant difference compared to the positive control (PC) group (SMS, 100 µg/mL); *, *p* < 0.05; **, *p* < 0.01, and ***, *p* < 0.001. -, not observed.

**Table 2:** Organic acid contents of CFS-*Lp*D25.

Organic Acid	Content (mg/mL)
Oxalic acid	0.174 ± 0.008
Tartaric acid	2.833 ± 0.065
Glycolic acid	ND
Ascorbic acid	ND
Malonic acid	2.206 ± 0.055
Lactic acid	12.493 ± 0.148
Pyroglutamic acid	0.762 ± 0.044
Succinic acid	2.618 ± 0.076
Fumaric acid	ND
Propionic acid	ND

Note: ND, not detected, below the minimum detection limit.

**Table 3:** API 50 CHL test results of *L. plantarum* D25.

Test	Active Ingredient	Sugar Classification	Fermentation Time (h)	
			24	48
GLY	Glycerol	Sugar Alcohol	-	-
ERY	Erythritol	Sugar Alcohol	-	-
DARA	D-Arabinose	Monosaccharide	-	-
LARA	L-Arabinose	Monosaccharide	+	+
RIB	D-Ribose	Monosaccharide	+	+
DXYL	D-Xylose	Monosaccharide	-	-
LXYL	L-Xylose	Monosaccharide	-	-
ADO	D-Ribitol	Sugar Alcohol	-	-
MDX	Methyl-ß-D-Xylopyranoside	Glycoside	-	-
GAL	D-Galactose	Monosaccharide	+	+
GLU	D-Glucose	Monosaccharide	+	+
FRU	D-Fructose	Monosaccharide	+	+
MNE	D-Mannose	Monosaccharide	+	+
SBE	L-Sorbose	Monosaccharide	-	-
RHA	L-Rhamnose	Monosaccharide	-	-
DUL	Galactitol	Sugar Alcohol	-	-
INO	Inositol	Sugar Alcohol	-	-
MAN	D-Mannitol	Sugar Alcohol	+	+
SOR	D-Sorbitol	Sugar Alcohol	+	+
MDM	Methyl-α-D-Mannopyranoside	Glycoside	+	+
MDG	Methyl-α-D-Glucopyranoside	Glycoside	-	-
NAG	N-Acetylglucosamine	Amino Sugar	+	+
AMY	Amygdalin	Glycoside	+	+
ARB	Arbutin	Glycoside	+	+
ESC	Aesculin, Ferric Citrate	Glycoside	+	+
SAL	Salicin	Glycoside	+	+
CEL	D-Cellobiose	Disaccharide	+	+
MAL	D-Maltose	Disaccharide	+	+
LAC	D-Lactose (Bovine Source)	Disaccharide	+	+
MEL	D-Maltotriose	Trisaccharide	+	+
SAC	D-Sucrose	Disaccharide	+	+
TRE	D-Trehalose	Disaccharide	+	+
NU	Inulin	Polysaccharide	-	-
MLZ	D-Melezitose	Trisaccharide	+	+
RAF	D-Raffinose	Trisaccharide	+	+
AMD	Starch	Polysaccharide	-	-
GLYG	Glycogen	Polysaccharide	-	-
XLT	Xylitol	Sugar Alcohol	-	-
GEN	Gentianose	Trisaccharide	+	+
TUR	D-Sophorose	Disaccharide	+	+
LYX	D-Lyxose	Monosaccharide	-	-
TAG	D-Tagatose	Monosaccharide	-	-
DFUC	D-Fucose	Monosaccharide	-	-
LFUC	L-Fucose	Monosaccharide	-	-
DARL	D-Arabitol	Sugar Alcohol	-	-
LAR	L-Arabitol	Sugar Alcohol	-	-
GNT	Potassium Gluconate	Sugar Acid Derivative	+	+
2KG	Potassium 2-Ketogluconate	Sugar Acid Derivative	-	-
5KG	Potassium 5-Ketogluconate	Sugar Acid Derivative	-	-

Note: + and - indicate the ability and inability to metabolize the carbohydrates, respectively.

**Table 4:** The MIC values of antibiotics against *L. plantarum* D25 and *S. aureus* ATCC 25923.

Antibiotic Drug	MIC (μg/mL)	
	*L. plantarum* D25	*S. aureus* ATCC 25923
FA	32	0.25
RIF	8	0.25
AMP	2	0.25
KAN	512	8
NEO	256	2
VAN	512	2
HYG	512	128

Note: FA, fusidate; RIF, rifamycin; AMP, ampicillin sodium; KAN, kanamycin; NEO, neomycin sulfate; VAN, vancomycin; and HYG, hygromycin hydrochloride.

## Data Availability

The whole genome sequence of *L. plantarum* D25 has been deposited in GenBank database under the accession number: GCA_055502195 (BioProject: PRJNA1399135, BioSample: SAMN54473911). The raw sequencing reads are available in the Sequence Read Archive (SRA) under the same BioProject accession PRJNA1399135.

## Supplementary Materials

The following supporting information can be downloaded at: https://www.mdpi.com/article/10.3390/foods15213951/s1. Table S1: The bacterial strains and media used in this study; Table S2: Information of the 53 *L. plantarum* genomes analyzed in the phylogenetic tree; Table S3: Sensory evaluation scoring criteria for shrimp meat samples; Table S4: Genome features of *L. plantarum* D25; Table S5 The predicted GIs in the *L. plantarum* D25 genome; Table S6 The predicted prophages in the *L. plantarum* D25 genome; Table S7: The predicted virulence determinants in the *L. plantarum* D25 genome; Table S8 The predicted ARGs in the *L. plantarum* D25 genome; Figure S1: The inhibition effect of CFS-*Lp*D25 on biofilm formation of *V. cholerae* GIM1.449; Figure S2: The hemolytic and gelatinase activity tests of *L. plantarum* D25. (A and B) The positive control *Staphylococcus aureus* ATCC25923 and *L. plantarum* D25 in the hemolytic activity test. (C and D) Before and after inoculation with a blank control (Tube 1), *L. plantarum* D25 (Tube 2), and *Staphylococcus aureus* ATCC25923 (Tube 3) in the gelatinase activity test; Figure S3: Agarose gel electrophoresis analysis of the plasmid DNA sample extracted from *L. plantarum* D25. Lanes 1 to 3: The plasmid DNA extract sample of *L. plantarum* D25, a positive control (plasmid pBD33), and a negative control (sterial ddH_2_O), respectively. Lane M: DNA molecular weight marker; Figure S4: Maximum-likelihood phylogenetic tree based on whole-genome sequences of 53 *L. plantarum* strains. The genome of *L. plantarum* D25 obtained in this study is highlighted with a red dot. The tree was constructed with 1,000 bootstrap replicates; Figure S5: The gene organization of the predicted GIs and prophages in the *L. plantarum* D25 genome. Genes were color-coded according to COG functional categories, and unknown genes are shown in white.
