# Supplementary material for: Lactiplantibacillus plantarum D25: A Promising Probiotic with Multi-Target Antibacterial Efficacy, Unique Metabolic Traits, Dual Thermal Sensitivity, and Biopreservative Potential for Refrigerated Shrimp
Source: Foods. 2026 Nov 3;15(21):3951. doi: 10.3390/foods15213951 (PMC13763325; doi:10.3390/foods15213951)
Supplement: Supplementary file 1 [file foods-15-03951-s001.zip › foods-4528597-supplementary.pdf]

Supplementary Tables

Table S1. The bacterial strains and media used in this study.

**Strain**	**Medium**	**Source**
Aeromonas hydrophila ATCC 35654	TSB	ATCC
Enterobacter cloacae ATCC 13047	TSB	BBBT
Enterobacter sakazakii CMCC 45401	TSB	BBBT
Escherichia coli ATCC 25922	TSB	ATCC
Klebsiella pneumoniae ATCC 43816	TSB	GIMCCC
Pseudomonas aeruginosa ATCC 27853	TSB	BBBT
Salmonella choleraesuis ATCC 13312	TSB	ATCC
Salmonella Paratyphi A GIM 1.235	TSB	GIMCCC
Salmonella typhimurium ATCC 15611	TSB	ATCC
Shigella dysenteriae ATCC 51252	TSB	GIMCCC
Shigella flexneri GIM 1.238	TSB	GIMCCC
Shigella sonnei GIM 1.239	TSB	GIMCCC
Staphylococcus aureus ATCC 25923	TSB	ATCC
V. alginolyticus ATCC 17749	TSB (3% NaCl)	ATCC
Vibrio alginolyticus ATCC 17749	TSB (3% NaCl)	ATCC
Vibrio cholerae GIM 1.449	TSB (3% NaCl)	GIMCCC
Vibrio harveyi ATCC BAA-1177	TSB (3% NaCl)	ATCC
Vibrio metschnikovii ATCC 700040	TSB (3% NaCl)	ATCC
Vibrio parahaemolyticus ATCC 17802	TSB (3% NaCl)	ATCC

Note: ATCC, American Type Culture Collection, USA; GIMCCC, Guangdong Institute of Microbiology Culture Collection Center, Guangzhou, China; and BBBT, Beijing Bio-Bridge Technology Co., Ltd., Beijing, China.

Table S2. Information of the 53 *L. plantarum* genomes analyzed in the phylogenetic tree.

No.	Strain	Assembly No.	Geographic location	Collected Year	Isolation Source
1	D25	GCA_055502195.1	China	2025	Dongbei kimchi
2	ER	GCF_001633245.1	France	2013	Adult
3	CNW10	GCF_001633285.1	USA	2013	Adult midgut
4	Nizo2757	GCF_001633485.1	Italy	2013	Sourdough
5	Nizo2814	GCF_001633575.1	Italy	2013	Wine grapes
6	Nizo2831	GCF_001633595.1	USA	2013	Grass silage
7	Nizo2256	GCF_001633805.1	France	2013	Stool
8	Nizo1838	GCF_001639425.1	France	2013	Stool
9	Nizo2259	GCF_001639545.1	France	2013	Tooth abscess
10	SRCM101060	GCF_001662895.1	South_Korea	2014	Kimchi
11	SRCM102022	GCF_002173655.1	South_Korea	2017	Food
12	K259	GCF_002868775.1	South_Korea	2017	Kimchi
13	PFC-311	GCF_003046075.1	Turkey	2017	Sourdough
14	SRCM103287	GCF_004054305.1	South_Korea	2018	Food
15	SRCM_103305	GCF_004055415.1	South_Korea	2018	Food
16	SRCM_103292	GCF_004055435.1	South_Korea	2018	Food
17	SRCM103295	GCF_004087995.1	South_Korea	2018	Food
18	SRCM103418	GCF_004101625.1	South_Korea	2018	Food
19	D31	GCF_004102885.1	China	2014	Pickle
20	SRCM103472	GCF_004103495.1	South_Korea	2018	Food
21	SRCM103473	GCF_004103515.1	South_Korea	2018	Food
22	SRCM103303	GCF_004141895.1	South_Korea	2018	Food
23	pc-26	GCF_006770485.1	China	2014	Healthy adult fecal
24	SRCM100995	GCF_009913675.1	South_Korea	2017	Pickle
25	SRCM101222	GCF_009913835.1	South_Korea	2017	Kimchi
26	SRCM101518	GCF_009913855.1	South_Korea	2017	Kimchi
27	L12	GCF_011421665.1	India	2014	Human feces
28	1001254B_1510	GCF_015556355.1	USA	2015	Stool
29	AR195	GCF_017351995.1	China	2014	Fermented product
30	XSBN-3	GCF_017963505.1	China	2020	Sour bamboo shoot
31	XSBN-10	GCF_017963525.1	China	2020	Sour bamboo shoot
32	T30PCM17	GCF_022568855.1	Italy	2013	Food
33	T30PCM31	GCF_022568865.1	Italy	2013	Food
34	T30PCM2	GCF_022568895.1	Italy	2013	Food
35	M4L1	GCF_023108905.1	China	2021	Preserved beancurd
36	CIRM-BIA_218	GCF_025212165.1	France	2016	Grape must
37	CIRM-BIA_218	GCF_025212205.1	France	2016	Grape must

38	GY3	GCF_026156675.1	China	2021	Pickle
39	S2.13	GCF_026156885.1	China	2021	Milk
40	XJSC	GCF_026156915.1	China	2021	Pickle
41	YC1.2	GCF_026156925.1	China	2021	Meat
42	XXS	GCF_026156985.1	China	2021	Pickle
43	SRCM101587	GCF_026976315.1	South_Korea	2017	Kimchi
44	Tw226	GCF_029997055.1	Argentina	2020	Intestinal tract
45	C36	GCF_030237845.1	China	2010	Milk
46	KCKM_0597	GCF_031348585.1	South_Korea	2016	Kimchi
47	KCKM_0112	GCF_031432955.1	South_Korea	2016	Kimchi
48	SN21-2	GCF_033802805.1	China	2019	Chicken
49	CQ01-107	GCF_049188355.1	China	2016	Fermented product
50	BE1003-8-8	GCF_051155255.1	USA	2010	Fermented product
51	BE1001-6-25	GCF_051155615.1	USA	2010	Fermented product
52	NIAB_GJ011C	GCF_051550575.1	India	2020	Jejunum
53	NIAB_GJ009C	GCF_051550615.1	India	2020	Jejunum

Table S3. Sensory evaluation scoring criteria for shrimp meat samples.

Evaluation Item	Score	Scoring Criteria Description
Color	5	Uniformly translucent appearance with high surface gloss, absence of discoloration or spots.
	4	Slight fading or dullness, good gloss, few localized spots.
	3	Dull or partially yellowish color, moderate gloss, visible minor
	2	Grayish or distinctly yellowish color, lacking gloss, presence of large spots or blackening.
	1	Severely blackened or yellowed, surface devoid of gloss, with
Odor	5	Pronounced characteristic fresh shrimp aroma, absence of
	4	Distinct fresh shrimp aroma, slight but acceptable off-odor.
	3	Weakened fresh shrimp aroma, presence of slight off-odor.
	2	No distinct fresh shrimp aroma, noticeable off-odor.
	1	Strong off-odor, completely unacceptable.
Texture	5	Clear muscle fiber texture, excellent elasticity (rapid recovery after pressing).
	4	Relatively clear muscle fiber texture, good elasticity (slower recovery after pressing).
	3	Slightly blurred texture, moderate elasticity (minimal recovery after pressing).
	2	Blurred texture, poor elasticity (significant deformation after
	1	Lacking elasticity, tissue soft or mushy (no recovery after
Overall Acceptability	5	Excellent overall quality, flawless in color, odor, and texture; highly acceptable to panelists.
	4	Good quality, minor defects do not affect overall acceptability.
	3	Moderate quality, presence of defects (e.g., in color or odor) but marginally acceptable.
	2	Poor quality, obvious defects, low acceptability.
	1	Very poor quality, completely unacceptable, disliked by panelists.

Table S4. Genome features of *L. plantarum* D25.

**Genome Feature**	***L. plantarum* D25**
Genome size (bp)	3,290,333
Clean reads	8, 362, 486
G + C (%)	44.44
DNA Scaffold	17
Total predicted gene	3077
Genes assigned to COG	2453
GI	8
Prophage	3
CRISPR-Cas	0
INT	0
IS	3
Genome accession No.	GCA_055502195.1

Note: GI, Genomic Island; CRISPR, Clustered Regularly Interspaced Short Palindromic Repeats; INT, Integrase; IS, Insertion Sequence.

Table S5. The predicted GIs in the *L. plantarum* D25 genome.

GI ID	Location	Length (bp)	Start (bp)	End (bp)	CDS Gene
GI 01	Scaffold 1	44421	137893	182314	55
GI 02	Scaffold 2	18841	301182	320023	14
GI 03	Scaffold 2	46816	508982	555798	44
GI 04	Scaffold 2	10708	582450	593158	9
GI 05	Scaffold 3	4642	322280	326922	7
GI 06	Scaffold 5	15772	23882	39654	13
GI 07	Scaffold 5	11737	76022	87759	13
GI 08	Scaffold 6	29017	222981	251998	28

Table S6. The predicted prophages in the *L. plantarum* D25 genome.

Prophage ID	Location	Length (bp)	Start (bp)	End (bp)	CDS Gene
Ph 01	Scaffold 1	31330	143923	175252	35
Ph 02	Scaffold 5	11066	66042	77107	15
Ph 03	Scaffold 8	40325	51850	92174	52

Table S7. The predicted virulence determinants in the *L. plantarum* D25 genome.

Gene_ID	Location	Gene Start	Gene End	Strand	VFDB ID	Vfs
gene_0111	Scaffold1	100299	101486	+	VFG046465(gb|WP_003028672)	EF-Tu (VF0460)
gene_0748	Scaffold2	120629	121549	+	VFG000964(gb|WP_010922799)	Hyaluronic acid capsule (VF0244)
gene_0778	Scaffold2	158547	157957	-	VFG000077(gb|NP_465991)	ClpP (VF0074)

Note: VFDB, Virulence Factor Database; Vfs, virulence factors.

Table S8. The predicted ARGs in the *L. plantarum* D25 genome.

Gene_ID	Location	Gene Start	Gene End	Strand	ARO Accession	Drug Class	Resistance Mechanism
gene_0111	Scaffold1	100299	101486	+	ARO:3003438	Elfamycin	Antibiotic target alteration
gene_0389	Scaffold1	379994	382000	+	ARO:3003314	Aminocoumarin	Antibiotic target alteration
gene_0976	Scaffold2	374184	377789	+	ARO:3007073	Peptide antibiotic;	Antibiotic target alteration/replacement
gene_0977	Scaffold2	377807	381448	+	ARO:3003291	Peptide	Antibiotic target alteration
gene_0979	Scaffold2	383045	383458	+	ARO:3003395	Aminoglycoside	Antibiotic target alteration
gene_0981	Scaffold2	384087	386183	+	ARO:3003735	Fusidane	Antibiotic target alteration
gene_0982	Scaffold2	387135	387443	+	ARO:3003930	Tetracycline	Antibiotic target protection
gene_2032	Scaffold5	30497	32441	+	ARO:3003301	Aminocoumarin	Antibiotic target alteration

Note: ARO, Antibiotic Resistance Ontology.

Supplementary Figures

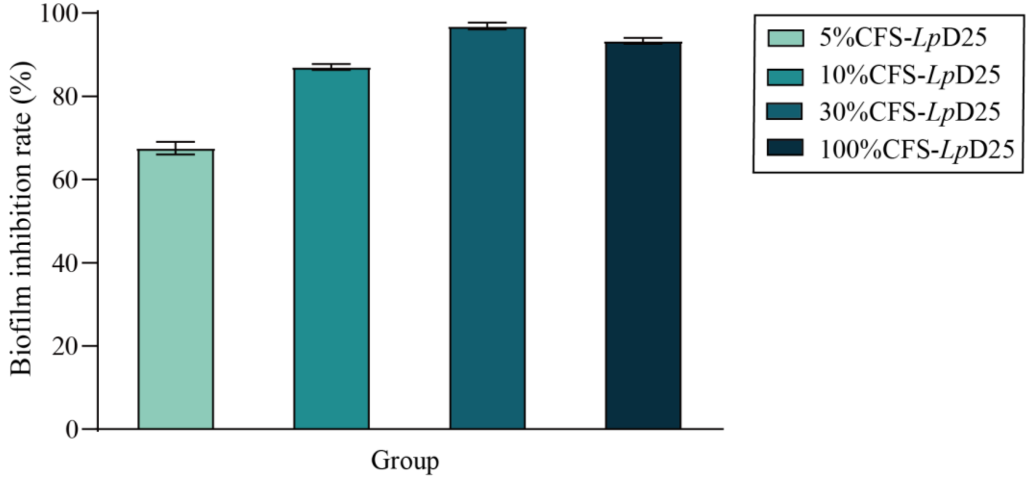

Figure S1.

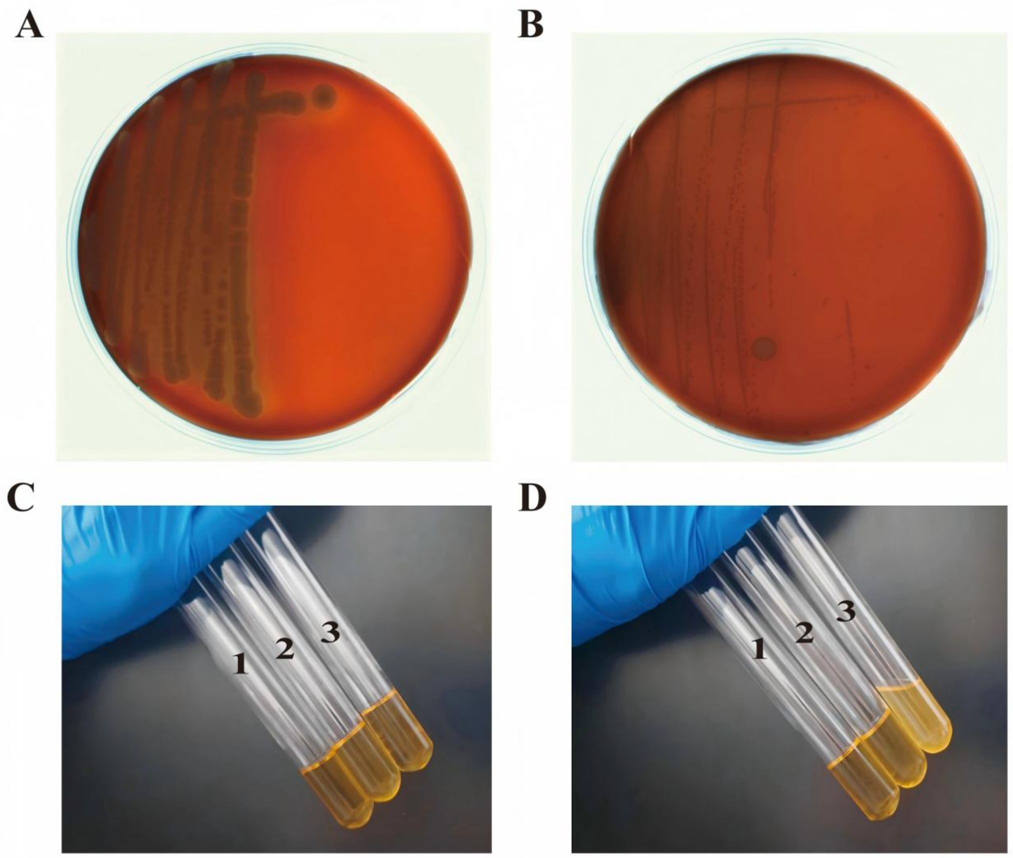

Figure S2.

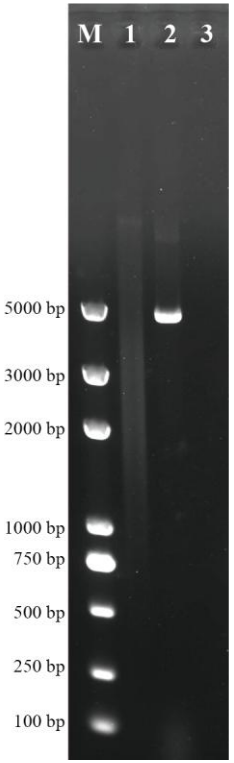

Figure S3.

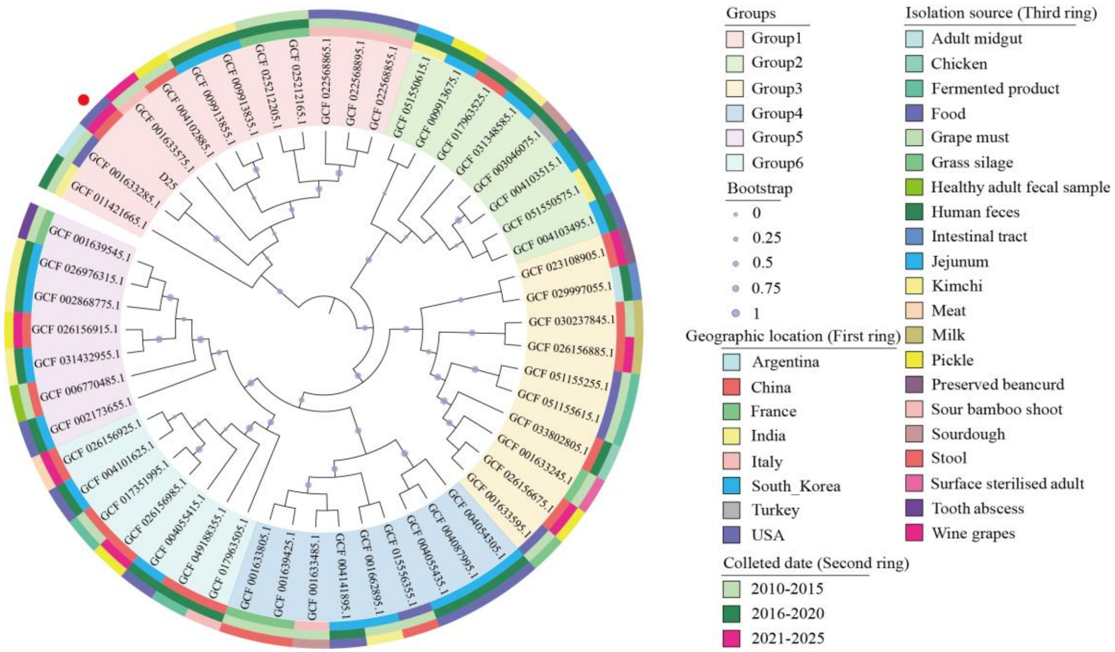
Groups
Group1
Group2
Group3
Group4
Group5
Group6
Bootstrap
0
0.25
0.5
0.75
1
Geographic location (First ring)
Argentina
China
France
India
Italy
South_Korea
Turkey
USA
Colleted date (Second ring)
2010-2015
2016-2020
2021-2025
Isolation source (Third ring)
Adult midgut
Chicken
Fermented product
Food
Grape must
Grass silage
Healthy adult fecal sample
Human feces
Intestinal tract
Jejunum
Kimchi
Meat
Milk
Pickle
Preserved beancurd
Sour bamboo shoot
Sourdough
Stool
Surface sterilised adult
Tooth abscess
Wine grapes

Figure S4

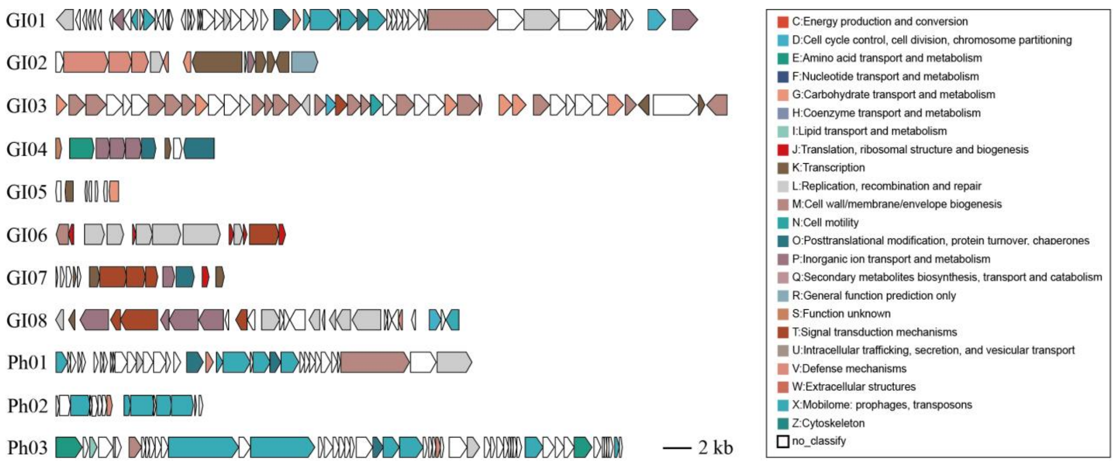
GI01
GI02
GI03
GI04
GI05
GI06
GI07
GI08
Ph01
Ph02
Ph03
2 kb
C:Energy production and conversion
D:Cell cycle control, cell division, chromosome partitioning
E:Amino acid transport and metabolism
F:Nucleotide transport and metabolism
G:Carbohydrate transport and metabolism
H:Coenzyme transport and metabolism
I:Lipid transport and metabolism
J:Translation, ribosomal structure and biogenesis
K:Transcription
L:Replication, recombination and repair
M:Cell wall/membrane/envelope biogenesis
N:Cell motility
O:Posttranslational modification, protein turnover, chaperones
P:Inorganic ion transport and metabolism
Q:Secondary metabolites biosynthesis, transport and catabolism
R:General function prediction only
S:Function unknown
T:Signal transduction mechanisms
U:Intracellular trafficking, secretion, and vesicular transport
V:Defense mechanisms
W:Extracellular structures
X:Mobilome: prophages, transposons
Z:Cytoskeleton
no_classify

Figure S5
